# The Adverse Effects and Use of Bevacizumab in Patients with Glioblastoma: A Systematic Review and Meta-Analysis

**DOI:** 10.3390/ph18060795

**Published:** 2025-05-25

**Authors:** Alejandro Bruna-Mejías, Vicente Silva-Bravo, Laura Moyano Valarezo, María Fernanda Delgado-Retamal, Diego Nazar-Izquierdo, Isidora Aguilar-Aguirre, Pablo Nova-Baeza, Mathias Orellana-Donoso, Alejandra Suazo-Santibáñez, Héctor Gutiérrez-Espinoza, Juan Sanchis Gimeno, Carlos Bastidas-Caldes, Juan José Valenzuela Fuenzalida

**Affiliations:** 1 Departamento de Ciencias y Geografía, Facultad de Ciencias Naturales y Exactas, Universidad de Playa Ancha, Valparaíso 2360072, Chile; alejandro.bruna@upla.cl; 2 Departamento de Morfología, Facultad de Medicina, Universidad Andrés Bello, Santiago 8370146, Chile; vicentesb2003@gmail.com (V.S.-B.); ldmoyanov@gmail.com (L.M.V.); fernanda.delgado1771@gmail.com (M.F.D.-R.); diego.nazar8@gmail.com (D.N.-I.); isiaguilar427@gmail.com (I.A.-A.); pablo.nova@usach.cl (P.N.-B.); mathor94@gmail.com (M.O.-D.); 3 Escuela de Medicina, Universidad Finis Terrae, Santiago 7501015, Chile; 4 Department of Morphology and Function, Faculty of Health and Social Sciences, Universidad de Las Américas, Santiago 8370040, Chile; alej.suazo@gmail.com; 5 Faculty of Education, Universidad Autónoma de Chile, Santiago 7500912, Chile; kineherctor@gmail.com; 6 GIAVAL Research Group, Department of Anatomy and Human Embryology, Faculty of Medicine, University of Valencia, 46001 Valencia, Spain; juan.sanchis@uv.es; 7 Facultad de Ingeniería y Ciencias Aplicadas, Biotecnología, Universidad de las Américas, Quito 170513, Ecuador; carlos.bastidas@udla.edu.ec; 8 Departamento de Ciencias Química y Biológicas, Facultad de Ciencias de la Salud, Universidad Bernardo O’Higgins, Santiago 8370993, Chile

**Keywords:** bevacizumab therapy, bevacizumab pharmaceutical, glioblastoma, multiform glioblastoma, glioma

## Abstract

**Background**: A glioblastoma (GBM) is a type of tumor originating from the glial brain cells, the astrocytes, and thus belongs to the astrocytoma group. Bevacizumab (BV) is a treatment for GBM. BV is the active ingredient in the drugs Avastin^®^, Alymsys^®^, Mvasi^®^ and ZiraBev^®^. It is currently approved as second-line treatment for GBM recurrence in combination with radiotherapy, and as first-line treatment for other cancers, including advanced colorectal cancer, metastatic breast cancer and advanced non-small-cell lung cancer. The objective of this systematic review was to analyze the scientific evidence from the science-based literature on the therapeutic effect and adverse effects of the drug BV in patients with GBM or GBM multiforme. **Methods**: We systematically searched electronic databases for the literature search, including the MEDLINE (via PubMed), SCOPUS, Google Scholar, the Cumulative Index to Nursing and Allied Health Literature and Web of Science databases, covering records from their earliest data to December 2024. Randomized or controlled clinical trials that were published in English or Spanish were included. The following keywords were used in different combinations: “Bevacizumab therapy”, “Bevacizumab pharmaceutical”, “Glioblastoma”, “Glioma” and “multiform glioblastoma”. **Results**: The use of Bevacizumab has been extensively studied in the scientific literature, with beneficial effects in symptom control. However, the adverse effects of BV vary across different types of carcinomas, which is why it has already been established that these adverse effects must be taken into consideration. In our meta-analysis of adverse effects, we found 14 adverse effects and estimated their prevalence, with an average of 19% (CI: 4 to 44%). The most significant vascular adverse effect was thromboembolism, which led to a greater number of complications for patients with GBM. Finally, the most common adverse effects were nausea, vomiting, fatigue and hypertension. **Conclusions**: While the beneficial properties of this pharmacological therapy have been observed, its adverse effect profile requires constant evaluation, as it includes vascular, blood and symptomatic adverse effects, which must be analyzed on a case-by-case basis and with great attention, especially in the case of more serious complications such as thromboembolic events.

## 1. Introduction

A glioblastoma (GBM) is a type of tumor originating from the glial brain cells, the astrocytes, and thus belongs to the astrocytoma group. In this group, GBM belongs to the highest level based on the WHO classification (grade IV), characterized as a primary malignant brain tumor that is aggressive, with a high degree of fatality, the worst survival prognosis among astrocytomas and an average life expectancy of 12 to 16 months, even with treatment. They typically occur in the central nervous system, and may be found in the brain, cerebellum, brainstem and spinal cord. The most common regions affected are the frontal or temporal lobes of the brain, without interhemispheric differences [1].

In addition, the development of metastasis has been described infrequently [2]. According to the literature, the prevalence of GBM varies between 0.59 and 5 cases per 100,000 people, with a slightly higher incidence in men, increasing with age [1]. A higher number of cases have also been reported in the Caucasian population, especially those living in industrial areas. A certain relationship and involvement of sex hormones and viral infections in the pathogenesis of the tumor has been identified [2]. In short, this pathology is on the increase, which may be linked to various factors, such as aging with higher life expectancy, air pollution and, on the other hand, the proliferation and improvement of neurological imaging techniques that allow for its diagnosis and overdiagnosis [1].

GBM is a spontaneously occurring neoplasm, and its progression is associated with cell cycle dysregulation at the G1/S checkpoint, leading to the presence of multiple genetic mutations in tumor cells [2]. Among the possible mutations, those associated with the enzyme isocitrate dehydrogenase (IDH) are particularly important, and, depending on the type of alteration in this enzyme, more or less aggressive clinical stages can occur. The IDH wild-type enzyme has a higher correlation with the violent progression of astrocytoma, in which progression is associated with the presence of astrocyte-like stem cells in the astrocytic band, located in the frontal and temporal lobes, the two regions most characteristic for the presence of GBM [1].

The complexity of GBM, in addition to its malignant and aggressive characteristics, includes the poor treatment options currently available, which contribute to the low survival rates of patients with this pathology.

Repeated mutations that occur at the onset of pathogenesis lead to the accumulation of growth factors that promote aberrant cell and tumor proliferation, such as vascular endothelial growth factor (VEGF), epidermal growth factor (EGF), platelet-derived growth factor (PDGF), hepatocyte growth factor (HGF) and the loss of phosphoenzyme analog (PTEN) [3]. In summary, these factors contribute to the key characteristics of GBM—marked neovascularization, increased mitotic activity, increment cellularity, nuclear pleomorphism and microscopic evidence of necrosis [4]. There are several drugs with different therapeutic targets that play a role in improving patient survival. One of the treatment approaches is targeted therapy, in which different pharmacological agents act on the receptors of the above-mentioned growth factors to inhibit and slow down the abnormal and accelerated proliferation of tumor cells. The group of drugs used in this approach includes those that inhibit the action of VEGF-A, a factor that promotes the formation of new blood vessels, such as Bevacizumab (BV) [3]. The treatment modalities and prognosis for glioblastoma (GBM) depend on tumor location, grade, genetic profile, proliferative activity, patient age and Karnofsky Performance Scale score [2]. However, the indication for adjuvant chemotherapy is assessed based on the patient’s clinical status and survival outlook, taking into account factors like age, quality of life as measured by the Karnofsky or ECOG scales, degree of tumor resection and mental status [5]. The standard treatment for a patient with primary GBM is the surgical resection of the tumor, followed by radiotherapy (RT) and concurrent chemotherapy (QT). Surgery serves both a diagnostic and therapeutic purpose and is fundamental to improving the prognosis of the patient, as it is recommended that the tumor lesion be resected as completely as possible while preserving the patient’s neurological function. Post-operative radiotherapy has been shown to increase patient survival and is therefore a key component of treatment; the current standard radiation algorithm for the treatment of GBM in non-frail patients involves a total dose of 60 Gy, delivered in 2 Gy fractions, one fraction per day, five days per week, for a total of 30 fractions. The concurrent use of QT is also important, with Temozolomide (TMZ) established as the first-line agent in 2020. TMZ is administered daily (75 mg/m^2^ per day) for 7 days during RT treatment, and approximately one month after the completion of RT with TMZ, 150–200 mg/m^2^ per day of TMZ is administered for 5 consecutive days every 28 days, for a total of six cycles. At present, BV is not approved as a first-line treatment for GBM. Although the combination of RT, TMZ and BV has not been shown to improve patient survival, it may extend the disease-free interval by approximately 3–4 months [6]. The use of BV has been suggested as one of the possible treatment options for the second-line treatment of GBM recurrence, still not defined. It has been approved for use in cases of high-grade glioma recurrence due to the increase in the disease-free interval [3,6,7,8,9].

The adverse effects associated with BV administration are of significant clinical relevance, as Bevacizumab is known to have a significant toxicity profile. Common clinical manifestations include high blood pressure, headache, dyspepsia, proteinuria, leukopenia, fatigue, stomatitis, epistaxis, dyspnea, upper respiratory tract infections and impaired wound healing. Less frequent but serious complications may include central nervous system hemorrhage, thromboembolic events, gastrointestinal perforation, left ventricular dysfunction and nephrotic syndrome [3,10]. Considering these potential adverse effects, concern and questions arise as to whether or not a patient with a de novo diagnosis of GBM truly benefits from treatment with BV. Preliminary data from articles state that there is a trend towards longer survival for patients with a low recursive partitioning technique (RPA) class compared to standard RT/TMZ treatment when BV is added, but not for those with a high RPA class [3].

The objective of this systematic review was to analyze the scientific evidence from the science-based literature on the adverse effects and therapeutic effects of the drug BV in patients with GBM.

## 2. Results

### 2.1. Study Selection

**Figure 1:**
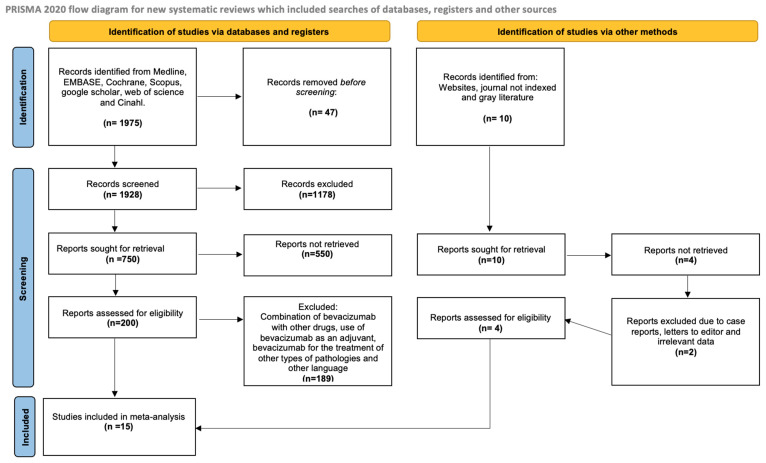
PRISMA flow diagram.

A total of 1975 studies were identified (Figure 1), accessed from the different databases with our established search terms. We excluded 47 studies that were not human studies, and then 1178 studies that did not mention the search terms in either their title or abstract were also excluded. This left 750 studies; of these, 550 did not report on the adverse effects of BV and only showed the use of BV, leaving 200 studies. Additionally, we eliminated 189 studies that combined BV with other drugs or used it as an adjuvant in treatment, studies in other pathologies, and finally, studies in languages other than English or Spanish, leaving a total of 11 studies; an additional 4 studies were identified through other search methods, bringing us to a total of 15 included studies. Ultimately, fifteen trials met the eligibility criteria and were included in this systematic review [11,12,13,14,15,16,17,18,19,20,21,22,23,24]. The kappa agreement rate between the reviewers was 0.88. The excluded studies and the reasons for their exclusion are available in Table S2 of the Supplementary Materials.

### 2.2. Study Characteristics

A summary of the included studies is presented in Table 1. The overall population included 2918 patients (1770 in the BV therapeutics group and 1148 in the other therapeutics groups). The mean age in the KLD group was 57.9 years (±3.1), the mean age in the other therapeutics group was 54.4 years (±4.1) and the mean follow-up duration was 90 days (ranging from 21 to 190) (Table 1).

### 2.3. Risk of Bias Assessment in Individual Studies and Heterogeneity

**Figure 2:**
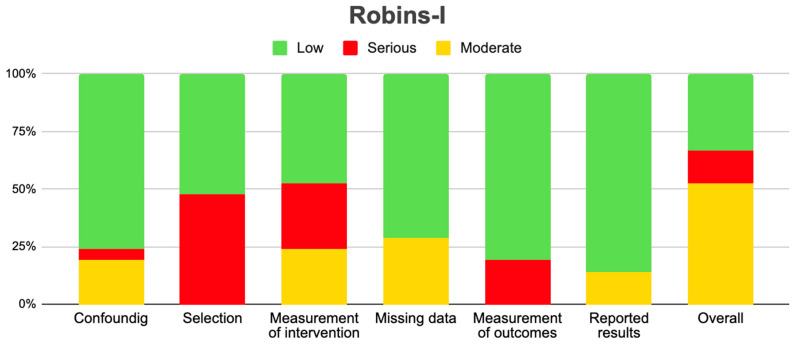
Risk of bias with Robbins I tools [11,12,13,14,15,16,17,18,19,20,21,22,26,27,28].

All studies were analyzed with the Robbins I, tool taking into account the six domains and the overall main risk factor for bias in the included studies. In relation to the above, two studies presented a serious overall risk of bias [15,17]. Regarding the domains among the fifteen articles analyzed, the domains that presented the highest risk of serious bias were sample selection and the blinding of the intervention (Table 2 and Figure 2).

When we went to analyze statistical heterogeneity in this study, there were specific risks of bias in some studies, so to see the prevalence in a more limited way, we performed subgroup analyses, giving the heterogeneity for each group and the analysis with the LFK index. Although heterogeneity has persisted in some analyses, we have made every effort to eliminate those heterogeneities that could be managed by our research group. Unfortunately, subgroup analysis carries its own difficulties, since when subgroups contain a low number of studies, they are prone to heterogeneity, which often causes the heterogeneity to persist. However, the analysis provides transparency to demonstrate that there was an adequate analysis. The use of random effects models in meta-analysis can help reduce heterogeneity and provide more accurate data, which is why the analysis was performed with this effect. Therefore, the prevalence of statistical heterogeneity is mainly related to the *n* and the proportion of subjects who had the condition.

### 2.4. BV with Another Drug vs. BV//BV with Another Drug vs. Another Drug

In compiling our findings, several studies compared treatment modalities involving Bevacizumab in the absence of or in conjunction with another drug, or vice versa; as such, the following is presented [11]. In 2021, a comparison between treatment with pembrolizumab as monotherapy versus pembrolizumab in concomitance with BV concluded that pembrolizumab with or without BV, despite good tolerability, where most adverse events were low-grade, was not effective for the treatment of recurrent GBM and had limited benefits for therapeutic use. The study in [23] analyzed the differences between the use of Temozolomide therapy with radiotherapy and BV with radiotherapy and irinotecan. The latter is notable for its higher median progression-free survival rate compared to TMZ, but there was no improvement in overall survival, which is associated with a possible high interaction rate. In addition, the use of BV + IRI did not alter patients’ quality of life compared to TMZ [18] in a comparison between BV alone and BV in conjunction with lomustine, revealing that the combination of low-dose BV plus lomustine was not superior to standard-dose BV in patients with recurrent GBM. However, a strong predisposition to better progression-free survival was observed in the group that received the low-dose BV in combination with lomustine. Field et al., 2015 [19], in their investigation of BV as monotherapy and its use in conjunction with carboplatin, described that they found no new adverse events beyond those already described in the literature. However, the addition of carboplatin implied a higher incidence of toxicity events without additional clinical benefit. Finally, they revealed that the clinical outcomes associated with the treatment of recurrent GBM with BV were inferior to those reported in previous studies. In contrast, ref. [19] conducted their study comparing the use of BV as monotherapy and BV with TVB-2640. Their findings indicated that there was a good oral tolerability of TVB-2640, along with its concomitant use with BV as biotherapy being safe for the patient, and showed that the overall response rate was 56% and that the progression-free survival rate was 31.4%, which showed a statistically significant improvement over the use of BV as monotherapy. Ref. [11], 2009, in a comparative analysis of BV as monotherapy and BV in combination with CPT-11 (irinotecan), concluded that both treatments were well tolerated and active against recurrent GBM. The adverse events in each treatment modality were similar to those reported in previous studies, and no new safety concerns were identified. Both treatments showed effective anti-tumor activity in patients with recurrent GBM, including tumor shrinkage in the majority of people. It is worth noting that the survival rate was significantly improved when CPT-11 was combined with BV, increasing from 15% to 49%; BV as monotherapy achieved a rate of 46.4%. Ref. [14], 2020, compared the use of BV as monotherapy with its use in combination with vorinostat. Although the combination of BV with vorinostat was well tolerated in terms of toxicity and side effects, it did not yield an improvement in progression-free survival, overall survival or clinical benefit compared to BV as monotherapy in adults with recurrent GBM. Moreover, patients reported moderate symptom interference with daily life in both modalities, and this interference increased over the course of the study in both modalities. Kickingereder et al., 2020 [23], studied two groups, one receiving BV with or without lomustine and the other treated with lomustine alone. Their results revealed that treatment with BV reduced tumor volume, angiogenesis and oxygenation, reflecting its efficacy in prolonging progression-free survival more than in the non-BV group. In spite of this fact, these parameters were not shown to be predictive of overall survival. Ref. [15], 2020, compared a BV-based treatment with nivolumab, both administered as monotherapy, and found that the median overall survival was comparable between the two modalities in the overall population with recurrent GBM. Furthermore, serious adverse events of any grade occurred at similar rates in both treatment groups. It is important to mention that, in the study, within tumor subcategories, patients with methylated tumors had a higher survival rate when treated with BV or nivolumab compared to patients with unmethylated tumors receiving the same treatment [16].

### 2.5. BV with Another Drug vs. Placebo

From the analysis of multiple trials that included placebo therapy, ref. [13], 2020, mentioned that, studying BV with trebananib versus BV with placebo, the former combination showed no significant improvement in survival, but the reason why those who received a BV and placebo therapy had better results were not clarified. Ref. [20] presented significant results for the use of placebo with chemoradiation for tumor volume reduction, overall survival and sustained tumor volume shrinkage compared to the use of chemoradiation with BV. Similarly, ref. [24] conducted a study comparing outcomes between the use of placebo and BV. They concluded that there was no significant difference in overall survival between the two regimens, but progression-free survival was longer in the BV group. Similarly, the first-line use of BV did not improve overall survival in patients with newly diagnosed GBM [24].

### 2.6. BV with Radiotherapy and Another Modality

Regarding the management of BV combined with radiotherapy, ref. [15] concluded that the combination of this drug with hypofractionated radiotherapy was able to improve progression-free survival, with a median length of 4.8 months with radiotherapy alone compared to an improvement to a median length of 7.6 months with the combined treatment, but overall survival had no significant difference, being only 0. However, an increase in PFS was more frequent in patients with a tumor of the RTK I methylation subtype and perineural gene expression, and it was concluded that molecular biomarkers such as perineural tumors and RTK I could better identify whether the patient would benefit from the combination of BV with hypofractionated radiotherapy or not. In a subsequent study by the same authors, Wirsching et al., 2021 [22], concluded that the combination of BV with RT improved survival in certain groups; this was the case for patients with greater volumes of tumors seen through contrast-enhanced MRI prior to treatment, improving their overall survival. The study further suggests that a higher apparent diffusion coefficient is associated with a greater overall survival benefit of the combined treatment. Additionally, a persistent FET-PET signal from a tumor in remission after concurrent treatment with BV is indicative of a pseudo-response and predicts a poor outcome. Importantly, the study by [28] is the only one that presents a direct comparison between RT + BV and the use of BV alone, while prior mentioned studies compared RT alone and RT combined with BV; this study by [28] fails to establish a significant overall survival benefit when combining BV with RT, with the overall survival length after combination therapy being 10.1 months compared to the survival of patients treated with BV alone, 9.7 months. However, in terms of progression-free survival at 6 months, there were differences between the two treatments, with a median length of 7.1 months for the combination therapy and 3.8 months for BV alone, leading the authors to finally conclude that the combination therapy alone can be considered safe and well tolerated with no related late toxicities [17,19,21,22,23,24].

### 2.7. Adverse Effects in GBM

With regard to adverse effects, data were compared for the following: diarrhea, which is a condition characterized by the presence of liquid stools or the need to defecate three or more times per day; hypophosphatemia, which is an insufficient amount of phosphorus in the blood within the normal range; nausea and vomiting, where nausea is the sensation of vomiting and vomiting is the unintentional expulsion of stomach contents; headache pain or discomfort in the cranial area, head, scalp or face; fatigue, which is a feeling of general fatigue, either a lack of energy or exhaustion; constipation, commonly known as obstipation, a condition in which the stool moves more slowly through the large intestine, making it harder and drier, accompanied by difficulty in evacuation; thrombocytopenia, a pathological decrease in the number of platelets in the blood; neutropenia, when the number of neutrophils in the blood falls below the normal range; lymphopenia, when the number of lymphocytes in the blood falls below the normal range; anemia, which is a condition characterized by a lack of sufficient amounts of healthy red blood cells or hemoglobin in the blood; and, finally, arterial hypertension, which is a condition that occurs when the blood pressure in the arteries is maintained at a high level for a prolonged period of time. When treating patients with BV alone, the most common adverse effects were headache, hypertension, constipation, fatigue and hypophosphatemia, while when BV was combined with other treatment modalities, the most common adverse effects were fatigue, headache, thrombocytopenia, nausea and vomiting and hypertension.

### 2.8. Adverse Event Statistics in GBM

Adverse events were analyzed across the included studies, and these data were then added together to obtain the overall study statistics. In the patients treated with BV alone, the following data were found: diarrhea occurred in 5% of patients, hypophosphatemia in 14%, nausea and vomiting in 9.2%, headache in 36.87%—being the most common adverse event in the whole study—fatigue in 24.1%, constipation in 29%, thrombocytopenia in 29%, thrombocytopenia in 14%, thrombocytopenia in 12.32%, neutropenia in 5.77%, lymphopenia in 9.32%, anemia in 2.19% and, finally, arterial hypertension, which was the second most common adverse event, with a prevalence of 32.72%.

When BV was combined with other treatment modalities, the following slightly different data were found: diarrhea occurred in 15% of patients, hypophosphatemia in 8.6%, nausea and vomiting in 13.37%, headaches in 15.65%, fatigue in 22.48%—being the second most prevalent adverse effect of the whole study—constipation in 23%—being the most prevalent adverse effect when combining BV with other treatments—thrombocytopenia in 14.83%, neutropenia in 10.2%, lymphopenia in 11.88%, anemia—being the least prevalent effect of the study—in 3.86% and, finally, hypertension at 13.2% prevalence (Table 3 and Table 4). 

### 2.9. Adverse Event Statistics Total

In order to better analyze the adverse effects of BV, studies of its implementation in other types of cancer were analyzed to compare the adverse effects of BV (individual prevalence in these studies shown in Table 5). Once these data were obtained, Table 3, Table 4 and Table 5 were constructed to obtain the global prevalence of the adverse effects generated by BV, both in glioblastoma and in other types of cancer, giving the following results: hypertension occurred in 19.3% of patients, leukopenia in 32.61%, anorexia in 31.91%, fatigue in 39.1%, proteinuria in 14%, diarrhea in 19.70%, anemia in 7.8%, headache in 19.7%, constipation in 20.7%, thrombocytopenia in 14.3%, lymphopenia in 11.4%, nausea and vomiting in 14.7%, hypophosphatemia in 11.30%, neutropenia in 12.4% and, finally, with the lowest prevalence but also the most serious side effect, thrombosis, with a rate of 4.17% [29,30,31,32,33,34,35,36,37,38,39].

#### 2.9.1. General Adverse Effects of Bevacizumab (In Other Types of Cancers)

The adverse effects reported in studies evaluating types of cancer other than GBM are as follows: diarrhea, nausea and vomiting, headache and fatigue. For hematological complications we have thrombosis—which is the formation of clots in the blood vessels that can cause serious problems such as embolisms—thrombocytopenia, neutropenia, lymphopenia, leukopenia—which is a decrease in the number of leukocytes in the blood—and anemia. The rest of the adverse effects are hypertension, proteinuria, which is the abnormal presence of proteins in the urine which may be an indication of kidney damage, anorexia which is the loss of appetite, and constipation. Their individual prevalence by study is detailed in Table 5.

The overall prevalence of these adverse effects, only evaluated in studies of cancers other than GBM, are as follows: diarrhea occurred in 24% of patients, anorexia in 32%, nausea and vomiting in 43.3%, headache in 50%, fatigue in 30%, thrombosis in 4.25%, thrombocytopenia in 26%, neutropenia in 47%, lymphopenia in 20%, leukopenia in 33%, anemia in 55%, hypertension in 26%, proteinuria in 13% and constipation in 21%.

#### 2.9.2. Arterial Hypertension

**Figure 3:**
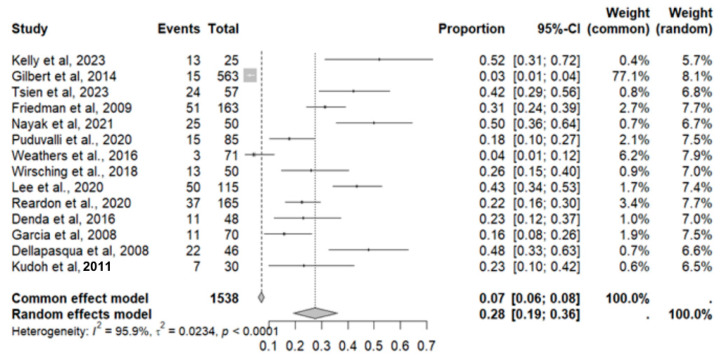
Forest plot of adverse effects (hypertension) [11,12,13,14,15,16,17,18,20,25,29,30,31,35].

**Figure 4:**
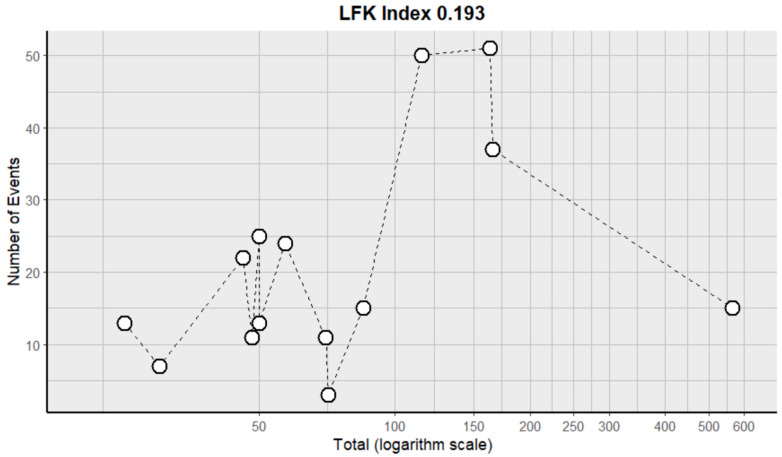
DOI plot of LFK index (hypertension).

This side effect refers to the fact that patients who consume VB may show an increase in their blood pressure levels despite not necessarily having an underlying condition of this pathology. For this adverse effect, fourteen studies were included that reported it, with a total number of included subjects of 1538 and a prevalence of 28% (CI: 19 to 36%), a heterogeneity (I^2^) of 95.9% and a *p* value = 0.0001 (Figure 3). For this prevalence, the DOI graph of the LFK index showed a value of 0.193 (Figure 4).

#### 2.9.3. Diarrhea

**Figure 5:**
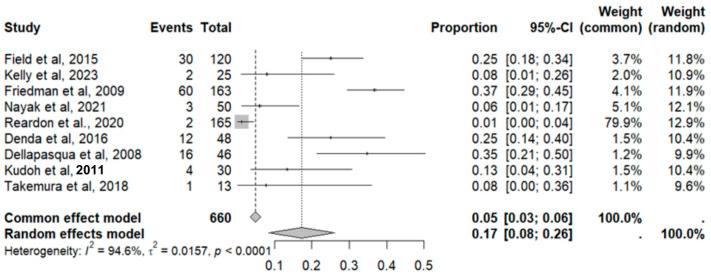
Forest plot of adverse effects (diarrhea) [11,12,16,19,20,29,31,35,39].

**Figure 6:**
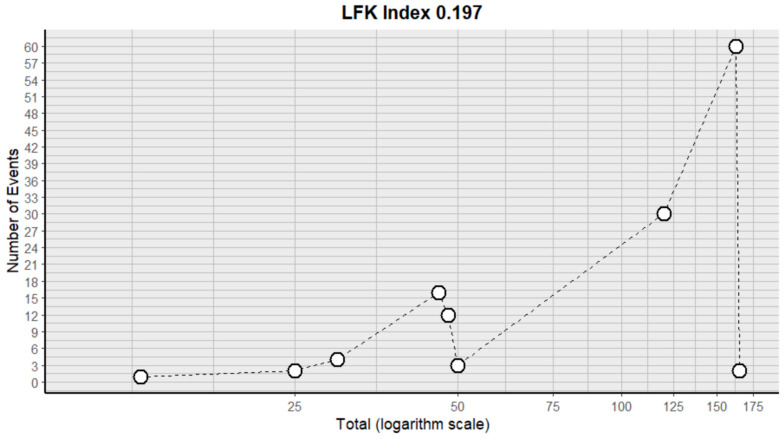
DOI plot of LFK index (diarrhea).

This side effect refers to the fact that patients who consume VB may experience the evacuation of loose or liquid stools more frequently than normal. For this adverse effect, nine studies were included that reported it, with a total number of included subjects of 660 and a prevalence of 17% (CI: 8 to 26%), a heterogeneity (I^2^) of 94.6% and a *p* value = 0.0001 (Figure 5). For this prevalence, the DOI graph of the LFK index showed a value of 0.197 (Figure 6).

#### 2.9.4. Headache

**Figure 7:**
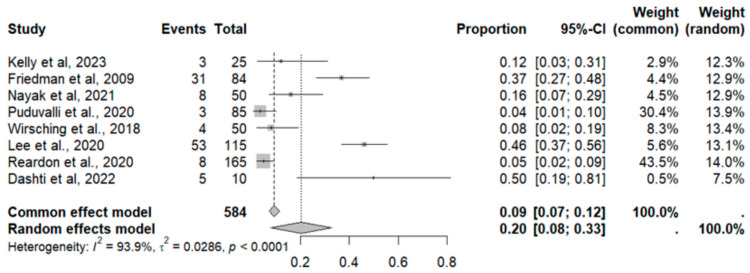
Forest plot of adverse effects (headache) [11,12,14,15,16,18,20,33].

**Figure 8:**
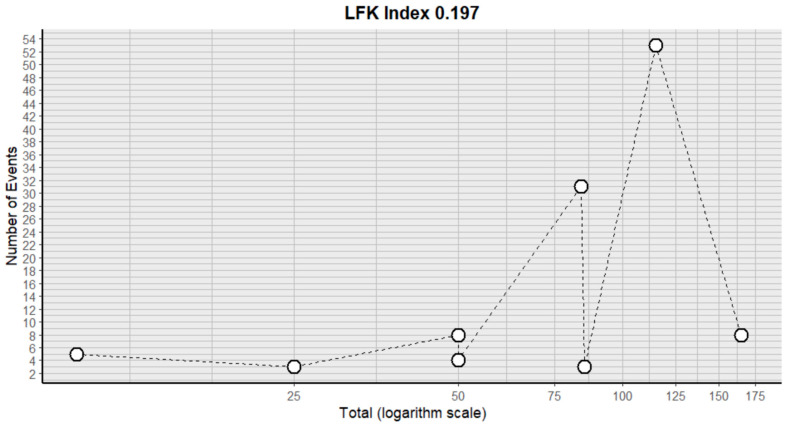
DOI plot of LFK index (headache).

This side effect refers to the fact that patients who consume VB may present headaches that can be felt in any part of the head, including the scalp, face and inside the head. For this adverse effect, eight studies were included that reported it, with a total number of included subjects of 584 and a prevalence of 20% (CI: 8 to 33%), a heterogeneity (I^2^) of 93.9% and a *p* value = 0.0001 (Figure 7). For this prevalence, the DOI graph of the LFK index showed a value of 0.197 (Figure 8).

#### 2.9.5. Fatigue

**Figure 9:**
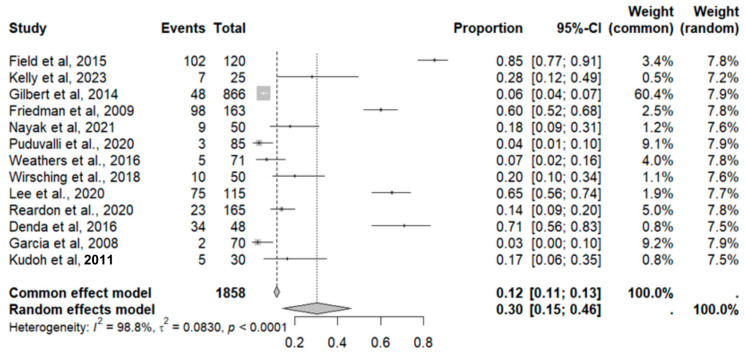
Forest plot of adverse effects (fatigue) [11,12,14,15,16,17,18,19,20,25,29,30,35].

**Figure 10:**
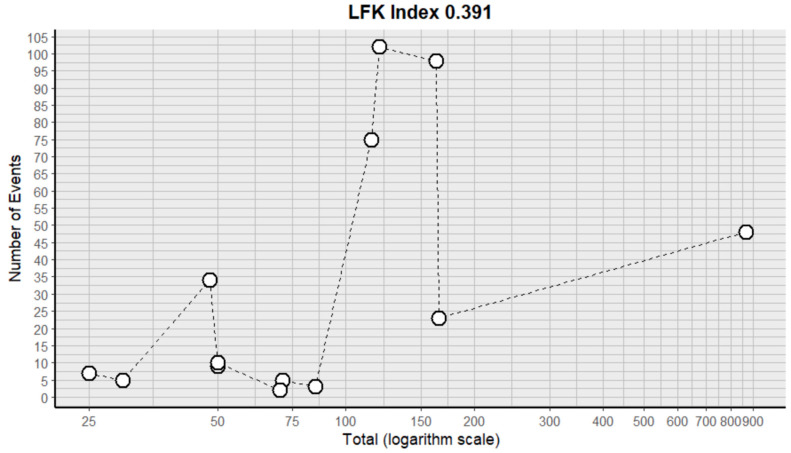
DOI plot of LFK index (fatigue).

This side effect refers to the fact that patients who consume VB may present feelings of tiredness and a lack of energy. For this adverse effect, thirteen studies were included that reported it, with a total number of included subjects of 1858 and a prevalence of 30% (CI: 15 to 46%), a heterogeneity (I^2^) of 98.8% and a *p* value = 0.0001 (Figure 9). For this prevalence, the DOI graph of the LFK index showed a value of 0.391 (Figure 10).

#### 2.9.6. Constipation

**Figure 11:**
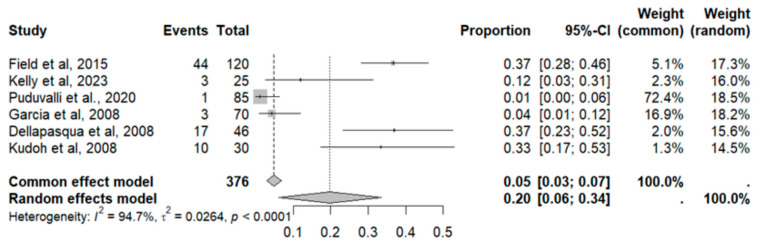
Forest plot of adverse effects (constipation) [15,19,20,30,31,35].

**Figure 12:**
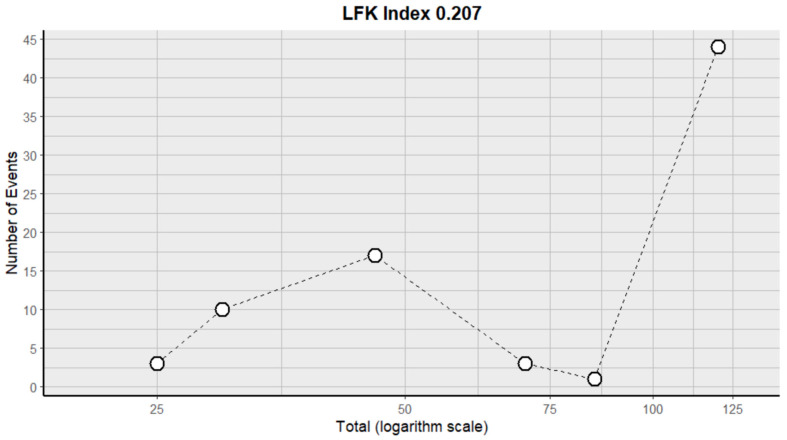
DOI plot of LFK index (constipation).

This side effect refers to the fact that patients who consume VB may present stools that are hard, dry and difficult to evacuate, and bowel movements that are less frequent and more painful. For this adverse effect, six studies were included that reported it, with a total number of included subjects of 376 and a prevalence of 20% (CI: 6 to 34%), a heterogeneity (I^2^) of 94.7% and a *p* value = 0.0001 (Figure 11). For this prevalence, the DOI graph of the LFK index showed a value of 0.207 Figure 12.

#### 2.9.7. Thrombocytopenia

**Figure 13:**
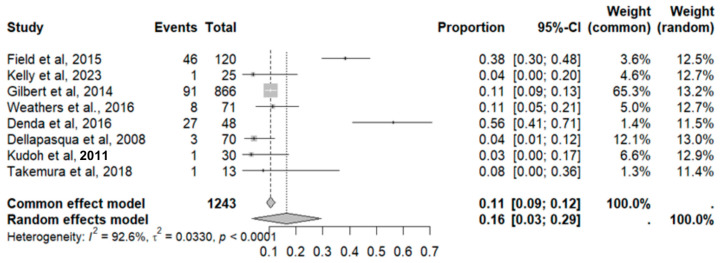
Forest plot of adverse effects (thrombocytopenia) [17,19,20,25,29,31,35,39].

**Figure 14:**
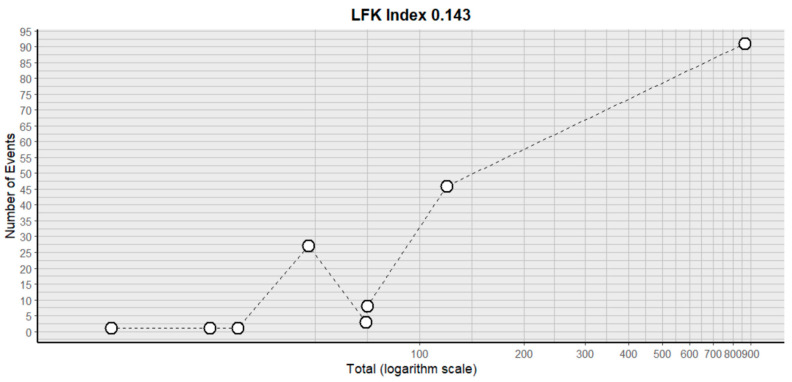
DOI plot of LFK index (thrombocytopenia).

This side effect refers to the fact that patients who consume VB may have lower-than-normal platelet counts in their blood. For this adverse effect, eight studies were included that reported it, with a total number of included subjects of 1243 and a prevalence of 16% (CI: 3 to 29%), a heterogeneity (I^2^) of 92.6% and a *p* value = 0.0001 (Figure 13). For this prevalence, the DOI graph of the LFK index showed a value of 0.143 (Figure 14).

#### 2.9.8. Neutropenia

**Figure 15:**
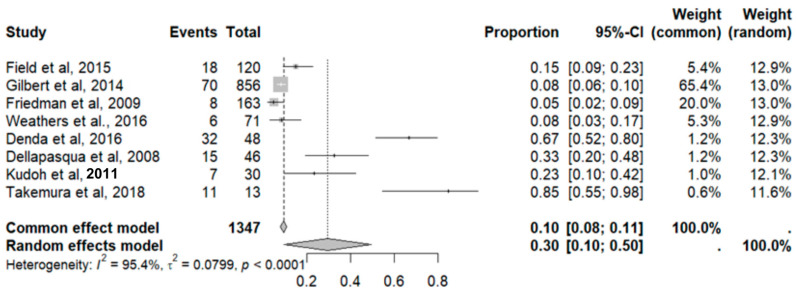
Forest plot of adverse effects (neutropenia) [11,17,19,25,29,31,35,39].

**Figure 16:**
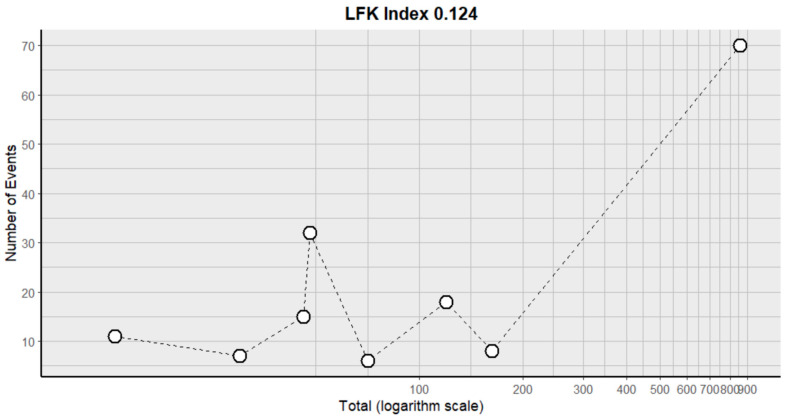
DOI plot of LFK index (neutropenia).

This side effect refers to the fact that patients who consume VB may have lower-than-normal amounts of neutrophils in their blood. For this adverse effect, eight studies were included that reported it, with a total number of included subjects of 1347 and a prevalence of 30% (CI: 10 to 50%), a heterogeneity (I^2^) of 95.4% and a *p* value = 0.0001 (Figure 15). For this prevalence, the DOI graph of the LFK index showed a value of 0.124 (Figure 16).

#### 2.9.9. Lymphopenia

**Figure 17:**
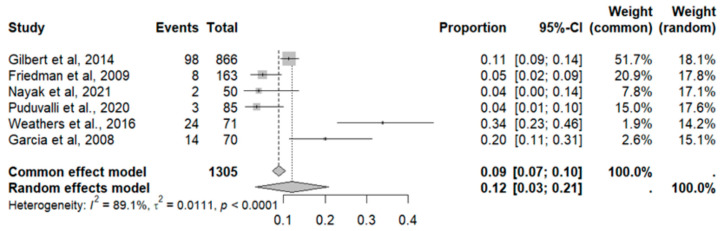
Forest plot of adverse effects (lymphopenia) [11,12,15,17,25,30].

**Figure 18:**
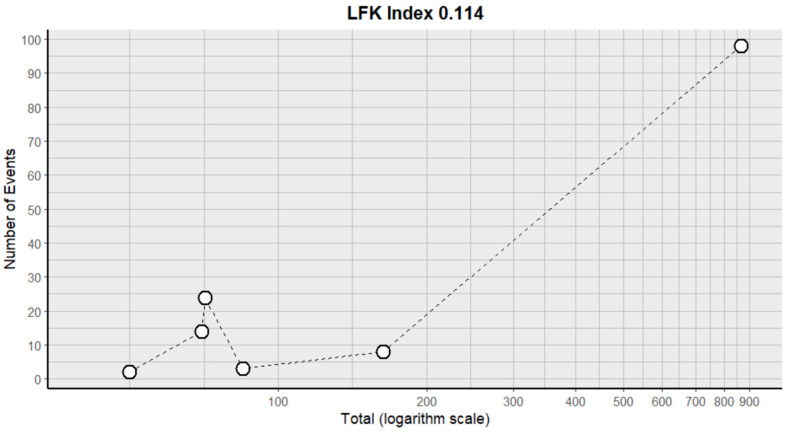
DOI plot of LFK index (lymphopenia).

This side effect refers to the fact that patients who consume VB may have lower-than-normal amounts of lymphocytes in their blood. For this adverse effect, six studies were included that reported it, with a total number of included subjects of 1305 and a prevalence of 12% (CI: 3 to 21%), a heterogeneity (I^2^) of 89.1% and a *p* value = 0.0001 (Figure 17). For this prevalence, the DOI graph of the LFK index showed a value of 0.114 (Figure 18).

#### 2.9.10. Nausea and Vomiting

**Figure 19:**
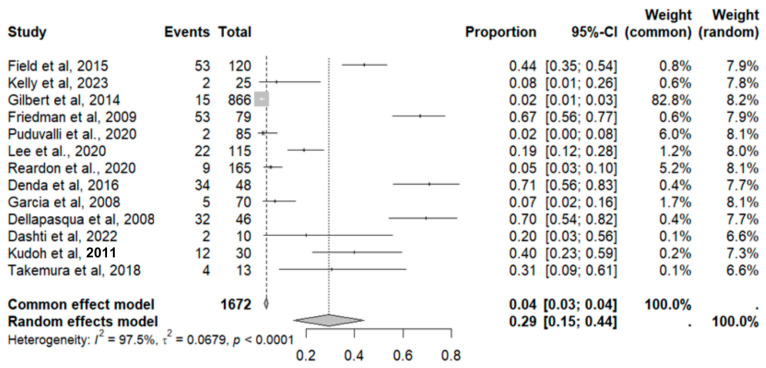
Forest plot of adverse effects (nausea and vomiting) [11,14,15,16,19,20,25,29,30,31,33,35,39].

**Figure 20:**
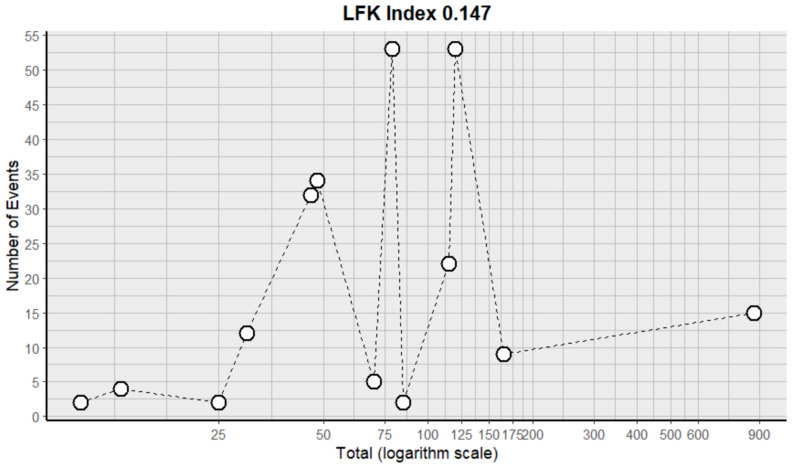
DOI plot of LFK index (nausea and vomiting).

This side effect refers to the fact that patients who consume VB may present nausea, which is the sensation of stomach discomfort, accompanied by vomiting, which is the expulsion of the contents of the stomach through the mouth. For this adverse effect, thirteen studies were included that reported it, with a total number of included subjects of 1672 and a prevalence of 29% (CI 9 to 61%), a heterogeneity (I^2^) of 97.5% and a *p* value = 0.0001 (Figure 19). For this prevalence, the DOI graph of the LFK index showed a value of 0.147 (Figure 20).

#### 2.9.11. Anemia

**Figure 21:**
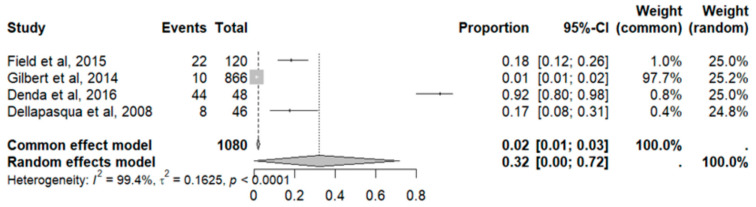
Forest plot of adverse effects (anemia) [19,25,29,31].

**Figure 22:**
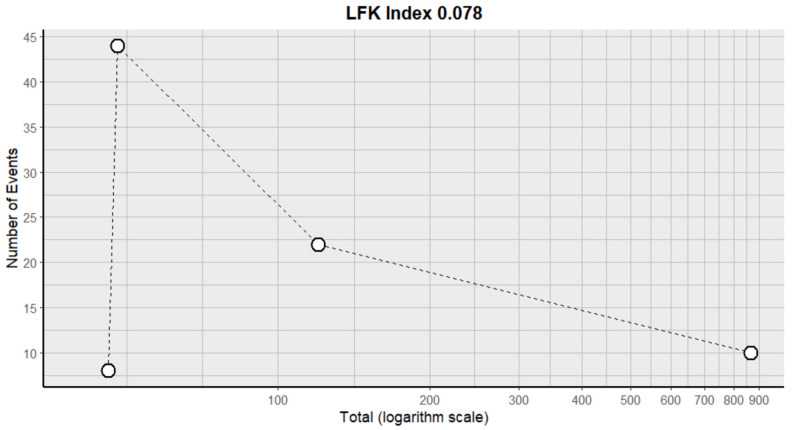
DOI plot of LFK index (anemia).

This side effect refers to the fact that patients who consume VB may have a lower-than-normal number of red blood cells or concentration of hemoglobin in their blood. For this adverse effect, four studies were included that reported it, with a total number of included subjects of 1080 and a prevalence of 32% (CI: 0 to 72%), a heterogeneity (I^2^) of 99.4% and a *p* value = 0.0001 (Figure 21). For this prevalence, the DOI graph of the LFK index showed a value of 0.078 (Figure 22).

#### 2.9.12. Anorexia

**Figure 23:**
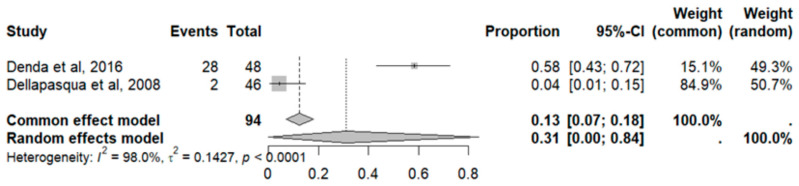
Forest plot of adverse effects (anorexia) [29,31].

**Figure 24:**
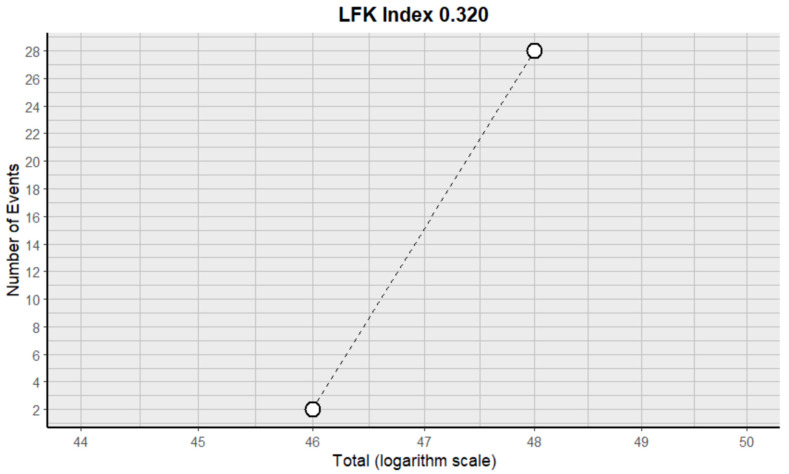
DOI plot of LFK index (anorexia).

This side effect refers to the fact that patients who consume VB may present a loss of appetite that is not caused by an eating disorder. For this adverse effect, two studies were included that reported it, with a total number of included subjects of 84 and a prevalence of 31% (CI: 0 to 84%), a heterogeneity (I^2^) of 98.0% and a *p* value = 0.0001 (Figure 23). For this prevalence, the DOI graph of the LFK index showed a value of 0.320 (Figure 24).

#### 2.9.13. Thrombosis

**Figure 25:**
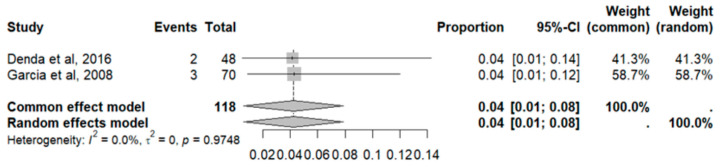
Forest plot of adverse effects (thrombosis) [29,30].

**Figure 26:**
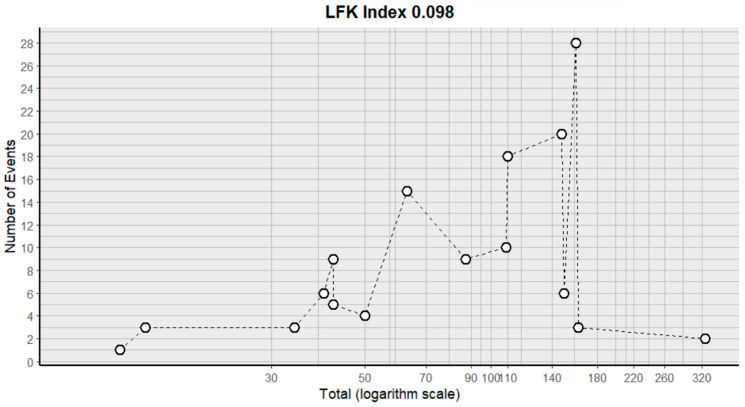
DOI plot of LFK index (thrombosis).

Thrombosis showed a prevalence of 4% (CI: 1 to 8%), with a heterogeneity (I^2^) of 0.0% and a *p* value = 0.0001 (Figure 25). For this prevalence, the DOI graph of the LFK index showed a value of 0.042 (Figure 26).

#### 2.9.14. Leukopenia

**Figure 27:**
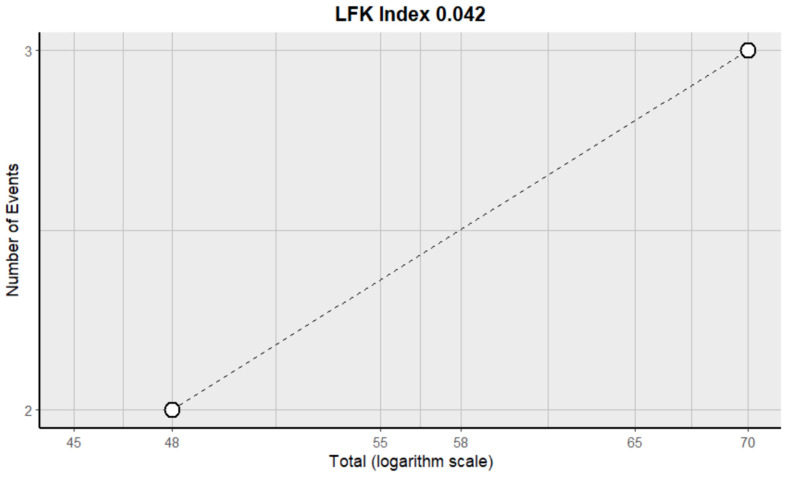
Forest plot of adverse effects (leukopenia).

**Figure 28:**
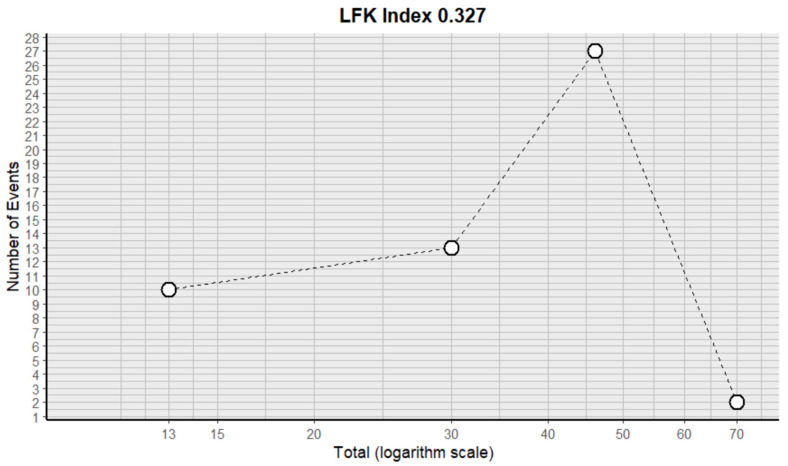
DOI plot of LFK index (leukopenia).

This side effect refers to the fact that patients who consume VB may have a lower number of leukocytes in their blood than normal. For this adverse effect, four studies were included that reported it, with a total number of included subjects of 159 and a prevalence of 44% (CI: 13 to 76%), a heterogeneity (I^2^) of 97.1% and a *p* value = 0.0001 (Figure 27). For this prevalence, the DOI graph of the LFK index showed a value of 0.327 (Figure 28).

#### 2.9.15. Proteinuria

**Figure 29:**
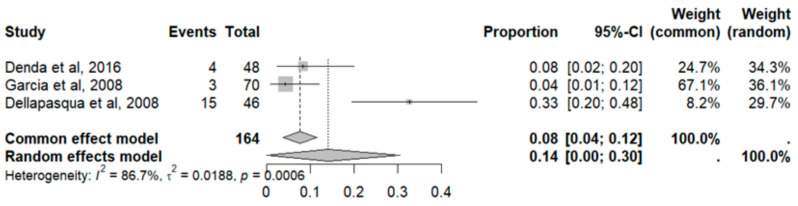
Forest plot of adverse effects (proteinuria) [29,30,31].

**Figure 30:**
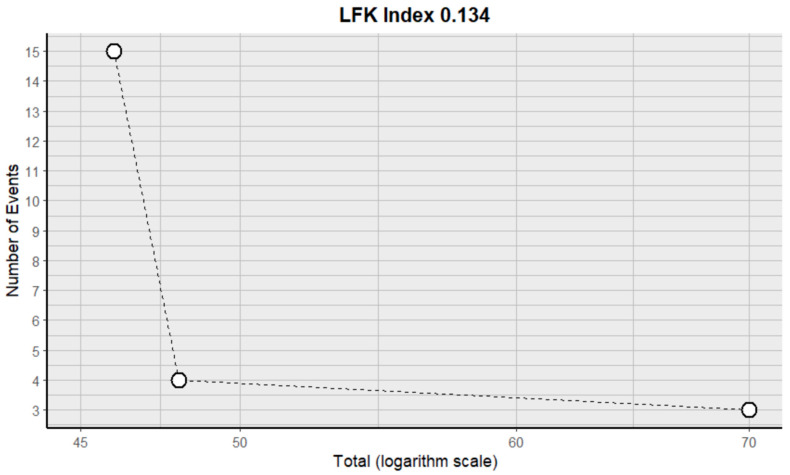
DOI plot of LFK index (proteinuria).

This side effect refers to the presence of protein in the urine in larger quantities than normal without previous kidney problems in patients who consume VB. For this adverse effect, three studies were included that reported it, with a total number of included subjects of 164 and a prevalence of 14% (CI 0 to 30%), a heterogeneity (I^2^) of 86.7% and a *p* value = 0.0001 (Figure 29). For this prevalence, the DOI graph of the LFK index showed a value of 0.134 (Figure 30).

## 3. Discussion

In this review, we have addressed the adverse effects and efficacy of BV in patients with GBM, showing that, although the effects are favorable in managing some symptoms in patients with GBM, which has been extensively described in the scientific literature, we have also shown that the adverse effects include more than 10 symptoms in patients with GBM, so the drug should be used with caution and clinicians should take into account that it could cause multiple effects associated with BV.

Regarding previous research on the effectiveness of using BV, we found four reviews that conducted research similar to our study. First, the study by Dongpo et al. (2022) [40], which aimed to explore the efficacy and safety of various Bev combination regimens in patients with recurrent GBM and to further explore the differences in the efficacy of each treatment in randomized controlled trials (RCTs) and non-randomized controlled trials (non-RCTs). The regimens examined were BV, BV + trebananib, BV + vorinostat, BV + lomustine, BV + carboplatin, BV + dasatinib, BV + irinotecan, BV + onartuzumab, BV + rindopepimut, BV + Temozolomide, lomustine, carboplatin, Procarbazine + lomustine + vincristine, Temozolomide, tumor treatment fields + BV, VB-111 and BV + VB-111, analyzed across 26 trials, 5 of which are also used in this review, resulting in a difference of 10 additional articles in the latter. The main conclusions mentioned by the authors were that BV + lomustine and BV + rindopepimut regimens could be considered effective for the treatment of recurrent GBM, in addition to the fact that the latter seemed to be safer and more effective. It was also shown that BV + irinotecan seemed to be effective according to a retrospective study. Finally, the difference between our study and the one presented lies in the fact that the latter only evaluated the total incidence of possible adverse effects, and did not provide a specific report on each of them with their respective incidence. Another review is that of Li, Huang and Xu (2015) [41], who performed a systematic review and meta-analysis with the aim of assessing the risk of cerebrovascular and other adverse vascular events in adult glioma patients receiving BV therapy. To do this, they included patients who received BV and those who were part of the control groups across four articles. None of these were consistent with the articles analyzed in this review. The authors reported as their main conclusions that BV treatment did not appear to significantly affect the risk of all-cause discontinuation, thrombocytopenia, deep vein thrombosis or pulmonary embolism in adult patients with newly diagnosed GBM. However, there was a trend towards the significance of BV treatment and the risk of pulmonary embolism. In addition, they recommend the use of anticoagulants in certain adult patients with newly diagnosed GBM who have a history of thromboembolism and/or more severe risk factors for thromboembolic events. Finally, the differences between their review and our study are that their paper specifically identifies cerebrovascular and vascular adverse events, rather than looking at the overall incidence of the different therapies, and also only evaluates the use of BV or control groups, rather than different combinations of modalities. Another study is that by Liu and Li (2024) [42], which aimed to clarify the survival prediction of apparent diffusion coefficient values in patients with recurrent GBM receiving BV treatment, analyzing various survival indicators, including overall survival and progression-free survival. Only patients who received BV as a treatment for recurrent GBM were included across the 10 studies analyzed in the meta-analysis. Of the included trials, only 1 was also used in this review, with a difference of 14 additional articles used for the development of the latter. The main conclusion proposed by those authors is that apparent diffusion coefficient values below the cut-off values could be associated with significant benefits for overall and progression-free survival compared with apparent diffusion coefficient values above the cut-off values. However, the bias caused by the different stages of recurrent GBM and different types and doses of BV regimens should not be ignored. Our study differs from the above study in that we evaluated the effects of different treatments for GBM, either according to the use of different drugs or therapeutic modalities, as well as the beneficial or detrimental interactions for the patient. Lastly, the study by Zhang (2012) [43] evaluated the efficacy and safety of BV compared to BV in combination with irinotecan for the treatment of recurrent multiform GBM. Therefore, the groups evaluated were patients who received BV alone and patients who received BV together with irinotecan. For this purpose, 11 trials were analyzed, 1 of which was also used in this review, with a difference of 14 additional articles being used for the development of the latter. The main conclusions drawn by the authors were that the combined treatment of BV with irinotecan produced effects at the imaging level that could be interpreted as an increase in progression-free survival, with no major effect on overall survival [32].

Otherwise, combination therapy is associated with toxicity, which hinders the adherence to and continuity of treatment, and there is also the economic factor, taking into account the significant cost savings of using BV as a single agent. Finally, an individual consideration for each patient is promoted, evaluating whether the combination of drugs provides benefits that outweigh the effects on treatment interruption and quality of life caused by drug toxicity. Our study differs from the studies above in that we consider more drugs and treatment modalities for GBM, as well as the different combinations between them. In terms of the clinical effects attributable to BV in relation to the studies analyzed, we report that this drug is considered as one of the possible therapeutic options for the treatment of GBM, which can be attributed to an increase in progression-free survival. In addition, the most common adverse events associated with its use are usually not serious, including headache, fatigue, constipation, hypertension and hypophosphatemia, all of which are ultimately treatable and manageable. It is important to note that the use of pembrolizumab, either as monotherapy or in combination with BV, although well tolerated by patients, has limited benefits without providing greater therapeutic benefit in cases of recurrent GBM. In contrast, a comparison between BV and nivolumab showed similar results in terms of median overall survival and serious adverse events. In summary, the combination of therapeutic modalities or drugs together with BV has been used in clinical practice or research for the treatment of GBM. It is important to study and discover the effects of the various interactions, as these will have different effects on patients and even on society. For example, the addition of carboplatin to a BV regimen has been shown to increase the incidence of toxic events without providing any additional clinical benefit. On the other hand, there are combinations of therapies that have shown no improvement and that would lead to increased medical costs without achieving better clinical outcomes. An example of this is the inclusion of the drug vorinostat in BV therapy, where, although it was well tolerated, it did not show an improvement in progression-free survival, overall survival or clinical benefit that would allow for its combined use to prevail over BV monotherapy. Similarly, the use of trebananib with BV did not show any significant improvements compared to the use of BV plus placebo. Although the above combinations are controversial in terms of their efficacy, there are therapeutic combinations that have a positive effect on the control of tumor progression, as well as on patient survival and quality of life. The different articles included in this review present guidelines, for example, for the combination of BV with irinotecan, lomustine, TVB-2640 and/or radiotherapy, which is associated with an improvement in progression-free survival. It has also been reported that, in certain cases, the combination of BV, radiotherapy and irinotecan did not alter quality of life and showed a better progression-free survival rate than the standard treatment for GBM, which consists of radiotherapy and Temozolomide. The main and most common adverse effects associated with a therapeutic combination that includes BV, characterized by the presence of fatigue, headache, thrombocytopenia, nausea and vomiting and/or arterial hypertension, must be taken into account when evaluating the appropriate and adequate treatment of patients [33,34].

The adverse effects reported in this meta-analysis were varied, with prevalences between 4 and 44%, with the lowest-percentage adverse effect being thrombosis, which the literature reports that patients with GBM have a higher risk of developing, regardless of their surgical, pharmacological or chemoradiation treatment. The presence of thrombosis does not affect survival. Tumor invasion of the dural sinuses and a higher fluid-attenuated inversion recovery ratio in pre-operative images were the most significant predictors of the development of thrombosis in patients with GBM; therefore, it does not have a direct relationship with the therapeutic use of BV [35]. Also, the most prevalent adverse effect of the use of BV was leukopenia, which is expressed as a decrease in the number of leukocytes (white blood cells) in the peripheral blood within the normal range. Generally, it is related to a decrease in the number of neutrophils, which is called neutropenia. A low neutrophil count is considered to be less than 1800 per microliter of blood [36]. This condition is not classic in patients with GBM, but it is directly attributable to the use of BV, so this should be taken into account in the future anamnesis of patients with GBM. Regarding the most reported adverse effects in the studies, these were nausea or vomiting, fatigue and hypertension. The former were reported in 12 to 14 studies each; for nausea or vomiting this adverse effect is one of the most present in the treatment of GBM, as most of the therapeutic options report it, so it is important to take it into account and inform patients. This adverse effect was shown in 29% of the studies, which indicates that approximately one in three patients will present with this adverse effect. Regarding fatigue, this was shown in 13 studies and showed a pooled prevalence of 30%; patients with GBM frequently present fatigue at the beginning of treatment, which suggests that other factors besides those related to radiotherapy, drugs or chemotherapy have a significant impact. In particular, depression and the location of the tumor could be specific factors for the presence of fatigue in these patients. This indicates that the main triggering factor for this condition is not the use of BV but rather factors that are more intrinsic to the pathology [37]. Regarding hypertension symptoms, clinically, this side effect presents as an increase of more than 10% of normal pressure. Since this could at least be normal in this type of patients, with respect to patients with GBM, for BV dosing, adjustments should be made so as not to increase blood pressure, which could bring adverse effects or important complications. In addition, we believe that the creation of validated scales that can analyze these factors would be necessary to quantify these conditions early [38]. A total of 14 studies included reports of this adverse effect, with a prevalence of 28%. Although this is an adverse effect, we have found literature suggesting that patients with glioblastoma who develop hypertension after treatment with BV show a significant improvement in overall survival compared to normotensive patients. These findings, the potential of hypertension as a biomarker of response to GBM treatment in individual patients and the role of hypertension as a modulator of interactions between tumor cells and cells in the perivascular niche make it a separate and emerging topic worthy of study. However, for this study, we understand that, although hypertension should be taken as a finding, an adverse effect, it has been shown to be favorable in the estimation of survival, which is why clinicians should take it into consideration and could even induce it [25,39].

Ultimately, it is important to consider the potential adverse events associated with BV treatment, with the aim of improving the quality of life and prognosis of patients. The most common adverse events are headache, constipation and high blood pressure in patients receiving BV as monotherapy, and constipation, fatigue and diarrhea in patients receiving combination therapy. In general, the adverse effects associated with the use of BV are similar whether it is used as monotherapy or in combination, but their prevalences differ to a greater or lesser extent. There is a high correlation between BV and high blood pressure, which may partly explain the high incidence of headache. Similarly, the presence of diarrhea and/or constipation may be explained by alterations in intestinal function, changes in mucous membranes and fluid and electrolyte losses, among other effects. Although these symptoms or signs, if severe and/or prolonged, could lead to the suspension of treatment, they are also events that can be overcome with pharmacological–dietary support, allowing treatment to continue without compromising the patient’s quality of life [25]. Since not all the studies considered presented adverse effects, we have only been able to group those that detailed them adequately, so there may be important adverse effects that have been omitted by the authors of the primary studies; therefore, although we provide an exhaustive analysis, we recommend that our results be analyzed with caution, and we also invite researchers to do more in-depth analyses that can better detail the adverse effects of BV in patients with GBM in primary studies [40,41,42,43,44,45,46,47,48,49,50].

In relation to the article by Brown et al. (2019) [51], which points out the difficulty in selecting documents due to the limitation of the use of keywords, even when applying MeSH terms, we followed their recommendations on the use of the RELISH database and turned to seed articles, which corresponded to the use of a key article or one that was representative of the topic to be investigated in our article. We used articles that included Bevacizumab and its adverse effects. This allowed us to enrich our databases with a greater amount of information and, therefore, identify a greater number of studies. Subsequently, as established by Brown et al. [51], the relevant articles were analyzed in order to identify which were the most useful and important for inclusion, and we proceeded to consider them for our research. Although they had already been found in the different databases collected, this reaffirmed that we did not have a large number of lost studies [51].

## 4. Materials and Methods

### 4.1. Protocol and Registration

This systematic review and meta-analysis were performed and reported according to the Preferred Reporting Items for Systematic Reviews and Meta-Analyses (PRISMA) statement [52]. The registration number in the International Prospective Register of Systematic Reviews (PROSPERO) is CRD4202222966.

### 4.2. Literature Search

We systematically searched electronic databases for the literature search, including the MEDLINE (via PubMed), SCOPUS, Google Scholar, the Cumulative Index to Nursing and Allied Health Literature and Web of Science databases, covering records from their earliest data to December 2024. Randomized or controlled clinical trials that were published in English or Spanish were included. The following keywords were used in different combinations: “Bevacizumab therapy”, “Bevacizumab pharmaceutical”, “Glioblastoma”, “Glioma” and “multiform glioblastoma”. Two authors (J.J.V.-F. and L.M.-V.) independently screened the titles and abstracts of the references retrieved from the searches. We obtained the full text for references that either author considered to be potentially relevant. We involved a third reviewer (M.F.) if a consensus could not be reached. To effectively locate studies on our topic, we used the detection in the biomedical literature search tool (https://relishdb.ict.griffith.edu.au/), accessed on 1 May 2025.

### 4.3. Study Selection

For the studies included in this meta-analysis, the following inclusion criteria were selected: patients with GBM or multiform GBM; patients who were administered BV at different doses; clinical symptoms of GBM, disability, symptom improvement and quality of life scales as evaluated outcomes; and study designs including randomized clinical trials, and experimental studies. Studies with the following characteristics were excluded: letters to the editor; reports/case series; reviews or non-human trials; studies that enrolled patients with other diseases; studies that administered therapies other than BV; and studies that did not have a control group as a comparator.

### 4.4. Data Extraction and Quality Assessment

The methodology of each study, as well as the outcomes of the studies and our outcomes, was measured using the Risk of Bias in Non-Randomized Studies of Interventions (ROBINS-I) tool [53] during data extraction. ROBINS-I offers a comprehensive set of tools to assess and decide on the risk of bias associated with confounding, the participant selection of trial participants, intervention measurements, deviations from the planned intervention, missing data, outcome measurements and reported results. The ROBINS-I tool can be used in non-randomized cross-sectional and longitudinal studies, as the quality is not unaffected by the study design. Based on the literature, confounding, age, ethnicity/race, physical activity and education were found to be the main confounders that needed to be appropriately adjusted in order for a study’s findings to be considered less risky. All areas were judged as being at a low, moderate, severe or critical risk of bias. Low risk indicated that a study was “like a successful randomized trial”, “in the area being evaluated”. A moderate risk of bias meant that the study was “good for a non-randomized study”, “but not up to par with or falls short of a full randomized trial”. If the risk of bias was very high, this meant that the risk of bias indicated that the study had “major problems”, “while if the risk of bias was very low, this meant that the risk of bias indicated that the study contained “too much information about the effects of the intervention”. If a domain did not contain enough information to determine whether the it had a risk of bias, then the domain was counted as having zero information. The total risk of bias for each article was the highest of all the assessed fields [53].

### 4.5. Statistical Methods

The data extracted from the meta-analysis was analyzed using R static software v.2025.05.0+496 (accessed in January 2025) to calculate the prevalence of the adverse effects BV. A DerSimonian–Laird model and a Freeman–Tukey double arcsine transformation combined the summary data. A random effects model was used due to the high heterogeneity observed in the prevalence data for the adverse effects BV. To assess the degree of heterogeneity among the included studies, the chi^2^ test and the I^2^ statistic were used. For the chi^2^ test, a *p* value of 0.10 was considered significant, as suggested by the Cochrane collaboration. The values of the I^2^ statistic were interpreted with a 95% confidence interval (CI) as follows: 0–40% indicated no significant heterogeneity, 30–60% suggested moderate heterogeneity, 50–90% reflected substantial heterogeneity and 75–100% denoted a considerable amount of heterogeneity [26]. To evaluate the presence of a small-study effect (the phenomenon that smaller studies may show different effects than large ones), a DOI plot of the LFK index results was generated [27,28].

## 5. Conclusions

A GBM is a tumor characterized by high malignancy and poor survival, with recent studies showing an average life expectancy of only 12–16 months, even among treated patients. For this reason, it is critical to find new therapeutic strategies or treatment combinations that can extend the survival of patients with GBM and enhance their quality of life in the short term. The use of BV has been studied as a promising alternative and has shown improvements in clinical parameters and progression-free survival, although, in some cases, without a significant impact on overall survival. While the beneficial properties of this pharmacological therapy have been observed, its adverse effect profile requires constant evaluation, as it includes vascular, blood and symptomatic adverse effects, which must be analyzed on a case-by-case basis with great attention, especially in cases of more serious complications such as thromboembolic events. To optimize the safety of BV, its combination with other therapeutic and/or pharmacological modalities should be considered, always on a case-by-case basis, to maximize the benefits and minimize the associated risks, in addition to taking into account adverse factors such as hypertension that could help patients with GBM. Therefore, future research should prioritize standardizing dosing regimens, exploring new combination therapies and finding ways to reduce and/or control the drug’s adverse effects, with the aim of improving both the prognosis and the quality of life of patients, and thus see if the survival of these patients can be increased over time.

### Limitations

This review was limited by the publication and authorship bias of the included studies. Furthermore, studies with different results that were in the non-indexed literature in the selected databases may have been excluded. Additionally, there could have been limitations in the sensitivity and specificity of the searches. Finally, the authors personally selected articles. All of these factors increment the probability of excluding potential cases from countries outside of Asia and North America that are not being reported by the scientific community.

## Figures and Tables

**Table 1:** Characteristics of included studies.

Author	Country	Population		Intervention		Outcomes	Follow-Up	Results
		Sample Size (*n*)	Patients’ Mean Age (SD)	Intervention	Characteristics/Dose			
Friedman et al., 2009 [11]	USA	CG: 82 EG: 85	CG: 57 EG: 54	CG: BV + CP-11 EG: BV	A duration of 6 months. All patients received BV 10 mg/kg intravenously every two weeks. Patients in the BV + CPT-11 group received CPT-11 340 mg/m^2^, if taking enzyme-inducing antiepileptic drugs [EIAEDs], intravenously for 90 min every two weeks. If not taking antiepileptic drugs, patients received 125 mg/m^2^ intravenously over 90 min every two weeks. No dose reduction in BV was allowed in case toxicity required the discontinuation of BV.	Objective response to medicine Partial response to medicine	2 months	Objective response to the drug: 28% of the BV group and 37.8% of the BV + CP-11 group. Partial response to the drug: 27.1% of the BV group and 35.4% of the BV-CP11 group.
Nayak et al., 2021 [12]	USA	CG: 30 EG: 50	CG: Not mentioned EG: Not mentioned	CG: pembrolizumab EG: pembrolizumab + BV	Durations of 4 months and two weeks. Pembrolizumab, 200 mg intravenously every 3 weeks, was administered as monotherapy. Pembrolizumab, 200 mg intravenously every 3 weeks, and BV, 10 mg/kg intravenously every 15 days, were administered as combination therapy.		48.6 months, Cohort A 49.6 months, Cohort B	Pembrolizumab alone or with BV was well tolerated, but its benefit was limited. The objective response rate was 20% and the median duration of response was 48 weeks.
Tsien et al., 2023 [13]	USA	CG: 86 EG: 84	CG: 60 EG: 57	CG: BV + RT EG: BV	The Re-RT dose was 35 Gy in 10 fractions, using 3D conformal RT, intensity-modulated RT or protons. BV was administered at a dose of 10 mg/kg once every 2 weeks. The initial cycle of BEV was initiated within 14 days of registration.		Not mentioned	Not mentioned
Lee et al., 2020 [14]	USA	CG: 58 EG: 57	CG: No average mentioned EG: No average mentioned	CG: BV + trebananib EG: BV + placebo	No previous treatment with GEFV inhibitors, including BV, or with angiopoietin-TIE2 inhibitors was allowed. All patients received BV at a dose of 10 mg/kg every two weeks. Trebananib was administered intravenously at a dose of 15 mg/kg every week until there was evidence of disease progression or severe toxicity associated with the treatment. The BV-only group was administered intravenously with placebo at a dose of 15 mg/kg.		6 months	The median survival time was 11.5 months. The median progression-free survival time was 4.8 months. The combination of trebananib and BV did not significantly improve the 6-month survival rate in patients with recurrent GBM at their first or second relapse compared to BV alone.
Puduvalli et al., 2020 [15]	USA	CG: 44 EG: 30	CG: No average is mentioned EG: No average is mentioned	CG: BV + Vor EG: BV	A duration of 48 weeks. A medical history and physical examination were performed at the beginning and at the beginning of each cycle. Complete blood counts were taken every 2 weeks. Serum biochemistry was measured every 4 weeks. A brain MRI and an MD Anderson Brain Tumor Symptom Inventory—Brain Tumor Survey (MDASI-BT) was completed by the patients every 8 weeks.	Progression-free survival General survival Number of relapses	19.84 months average	The patients in each group reported similar symptom burdens at baseline between the BV and BV plus vorinostat groups. In this study, the combination of vorinostat and BV was not superior to BV alone. Progression-free survival: P = 0.9401 BV
Reardon et al., 2020 [16]	USA	CG: 182 EG: 165	CG: 55.2 EG: 55	CG: Nivolumab EG: BV	A duration of 18 months. Patients received 3 mg/kg nivolumab intravenously every 2 weeks.		9.5 months	There was no statistical difference in the risk of death between the groups
Weathers et al., 2016 [17]	USA	CG: 33 EG: 36	CG: No average is mentioned EG: No average is mentioned	CG: BV + lomustine EG: BV	Treatment lasted 4.34 months for BV + lomustine and 4.11 months for BV. For the BV alone group, BV was administered intravenously at a dose of 10 mg/kg every 2 weeks until disease progression or unacceptable toxicity. In the combination group, BV was administered intravenously at a dose of 5 mg/kg every 3 weeks. There were 27 treatment cycles. The dose of lomustine could be reduced by a maximum of 2 times.		6 months	For the 12 patients treated with lomustine at a dose of 90 mg/m^2^ in combination with low-dose BV, there were 7 grade 4 hematological adverse events and 17 grade 3 events. The median survival was not significantly longer in the low-dose BV plus lomustine group.
Wirsching et al., 2018 [18]	Suiza	CG: 25 EG: 50	CG: 70 EG: 70	CG: B (only radiotherapy) EG: A (radiotherapy + BV) See radiotherapy	A duration of 10 months. Radiotherapy was administered to the gross tumor volume plus a 2 cm margin for 3 weeks. The radiotherapy included 15 fractions of 2.66 Gy, up to a total of 40.0 Gy. BV was administered intravenously at 10 mg/kg of body weight every 2 weeks.		Not mentioned	More severe and life-threatening thromboembolic events occurred in group A, with one death due to pulmonary embolism and one death due to myocardial infarction. The median survival without deterioration from the baseline was 5.7 months in arm A and 2.8 months in arm B.
Field et al., 2015 [19]	Australia	BV + ICE: 58. BV: 62.	BV + Carboplatin: 55. BV: 55.	BV + carboplatin BV	Doses of BV 10 mg/kg IV were administered every 2 weeks, plus ICE AUC 5 every 4 weeks. Monotherapy with BV 10 mg/kg IV administered every 2 weeks. The studied therapies continued until disease progression, unacceptable toxicity, participant withdrawal, non-compliance with protocol guidelines or death. The median follow-up was 32 months and the median treatment time was 3.3 months.	None	32 months	The addition of ICE resulted in increased toxicity without additional clinical benefit. For those treated with BV, the results were inferior to those in previously reported studies.
Kelly et al., 2023 [20]	USA	EG: 60	EG: 59	A1: TVB-2640 + BV. A2: BV.	BV monotherapy was administered alone for cycle 1 (28 days) for biomarker analysis. Thereafter, all patients received TVB-2640 plus BV until they reached treatment-related toxicity or disease progression. C1, D1 and D15 consisted of a dose of BV at 10 mg/kg IV. TVB-2640 mg/m^2^ was administered orally once daily. After completing C1, all patients received BV and TVB-2640 in combination in these same dosing regimens.	None	Not mentioned	TVB-2640 was a well-tolerated oral drug that could be safely combined with BV.
Ellingson et al., 2018 [21]	USA	CG: 459 EG: 458	CG: 56. EG: 55.	CG: Chemoradiation + placebo. EG: Chemoradiation + BV See radiotherapy	After undergoing surgical resection or biopsy, patients underwent concurrent RT and oral TMZ (75 mg/m2 of body surface area per day for up to 49 days) along with intravenous BV (10 mg/kg of body weight) or placebo every 2 weeks	Post-chemoradiation failure vs. disease control Post-surgical tumor volume Treatment (placebo vs. BV) Age KPS MGMT status	Not mentioned	A decrease in tumor volume during chemoradiation was associated with longer overall survival in the placebo group but not in the BV-treated group. Superior overall survival was observed in patients in the placebo group, with a sustained decrease in tumor volume.
Wirsching et al., 2021 [22]	Switzerland	BV + RTH: 44 RTH: 23	BV + RTH: 70 RTH: 69	BV + RTH RTH	Hypofractionated RT of 15 × 2.66 = 40 Gy in combination with BEV 10 mg/kg of body weight was administered every two weeks, compared with hypofractionated radiation therapy alone in elderly patients (>65 years) with newly diagnosed GBM. Molecular analyses were performed	Contrast enhancement (per cm^3^) ADC in contrast-enhancing tumor portion: high vs. low Alto vs. bajo 18FET TBR in contrast-enhanced tumors Portion: high vs. low.	4 months	A greater benefit of BV + RTH was observed compared to radiation therapy alone.
Kickingereder et al., 2020 [23]	Germany	CG: 93 EG: 161	CG: 60 EG: 58	BV groups: BV/BV + lomustina NO-BV group: Lomustina	A duration of 50 months. Magnetic resonance imaging analysis was conducted at the beginning and every 6 weeks until week 24, and then every 3 months. The study evaluated the optimal treatment sequence of BV and lomustine (four treatment arms with single-agent vs. sequential vs. combination treatment), while the subsequent phase III trial (two treatment arms) compared patients treated with lomustine alone with patients who received a combination of lomustine and BV (randomized in a 1:2 ratio).	Tumor volume with enhanced contrast Volume of FLAIR T2 signal abnormalities without contrast Normalized relative cerebral blood volume according to Gauss (nrCBF) Gaussian-normalized tumor metabolic rate of oxygen (nTMRO2)	Not mentioned	Progression-free survival was longer in the BV group compared to the NO-BV group. The volumes of T2 FLAIR signal abnormalities with and without contrast decreased in the BEV group, while they increased in the non-BEV group. None of the evaluated MRI parameters were predictive of overall survival in the BEV group. BV treatment reduced tumor volumes, angiogenesis and oxygenation, reflecting its efficacy in prolonging progression-free survival; however, these parameters were not predictive of overall survival (OS), highlighting the challenges of identifying patients who would benefit from OS with BV.
Herrlinger et al., 2016 [24]	Germany	CG: 54 EG: 116	CG: 56 EG: 56	CG: TMZ + RT EG: BV + RT + IRI	All patients received radiotherapy to the affected field from day 22 to 35 after surgery. In the experimental group, patients received BEV (10 mg/kg every 2 weeks), starting within the first week of RT but no earlier than day 28 after surgery, followed by maintenance BEV (10 mg/kg every 2 weeks) plus IRI (125 mg/m^2^ every 2 weeks; with enzyme-inducing antiepileptic drugs at a dose of 340 mg/m^2^ every 2 weeks). Second-line therapy was at the physician’s discretion.	C30 physical operation C30 global health C30 cognitive functioning BN20 motor dysfunction BN20 communication deficit KPS Miniature mental state examinations	12 weeks	BV + IRI resulted in a higher rate compared to TMZ. However, BV + IRI did not improve overall survival. BV + IRI did not alter the patients’ quality of life compared to TMZ.
Gilbert et al., 2014 [25]	USA	CG: 309. EG: 312.	None	CG: Placebo EG: BV	Patients underwent radiotherapy treatment and daily Temozolomide treatment (75 mg per square meter of body surface area). The treatment with the placebo or BV began in week 4 of radiotherapy and continued for up to 12 cycles of maintenance chemotherapy.	Neurological function MGMT status Molecular profile RPA class	Patients evaluated weekly for adverse events had weekly complete blood counts and monthly blood chemical analyses during radiotherapy During maintenance phase of treatment, patients underwent hemograms and blood chemical analyses on days 21 and 28 of each cycle Imaging studies of tumors	There were no significant differences in overall survival duration between the BV group and the placebo group. The progression-free survival time was longer in the BV group. Over time, greater symptom burdens, poorer qualities of life and declines in neurocognitive function were more frequent in the BV group. Modest increases in the rates of hypertension, thromboembolic events, intestinal perforation and neutropenia were observed in the BV group.

BV: Bevacizumab; CP-11: irinotecan; RT: radiotherapy; re-RT: reirradiation; pembrolizumab: monoclonal antibody directed against PD-1 surface protein; Cohort A: pembrolizumab + BV; Cohort B: pembrolizumab; ICE: carboplatin; TVB-2640: Denifanstat; A1: arm 1; A2: arm 2; RTH: hypofractionated radiotherapy; TMZ: Temozolomide; IRI: irinotecan; trebananib: kinase inhibitor; nivolumab: immune checkpoint inhibitor; VOR: vorinostat or suberoylanidide hydroxamic acid; lomustine: nitrosourea-type alkylating compound; KPS: Karnofsky Performance Scale; MGMT: O-methylguanine-DNA methyltransferase; RPA: Recursive Partition Analysis.

**Table 2:** Risk of bias assessed with Robbins I tools.

Author	Confounding	Selection	Measurement of Intervention	Missing Data	Measurement of Outcomes	Reported Result	Overall
Friedman et al., 2009 [11]	Low	Serious	Serious	Moderate	Low	Low	Moderate
Gilbert et al., 2014 [25]	Low	Low	Low	Low	Low	Low	Low
Field et al., 2015 [19]	Low	Serious	Serious	Moderate	Low	Low	Moderate
Herrlinger et al., 2016 [24]	Low	Low	Low	Low	Low	Low	Low
Weathers et al., 2016 [17]	Moderate	Low	Low	Low	Low	Low	Low
Ellingson et al., 2018 [21]	Low	Serious	Serious	Moderate	Low	Low	Moderate
Wirsching et al., 2018 [18]	Low	Low	Low	Low	Low	Low	Moderate
Kickingereder et al., 2020 [23]	Moderate	Serious	Moderate	Low	Serious	Moderate	Serious
Lee et al., 2020 [14]	Moderate	Serious	Serious	Moderate	Low	Low	Moderate
Puduvalli et al., 2020 [15]	Serious	Serious	Serious	Moderate	Low	Low	Serious
Reardon et al., 2020 [16]	Low	Low	Low	Low	Low	Low	Low
Wirsching et al., 2021 [22]	Low	Serious	Moderate	Low	Serious	Moderate	Moderate
Nayak et al., 2021 [12]	Low	Serious	Serious	Moderate	Low	Low	Moderate
Kelly et al., 2023 [20]	Low	Low	Low	Low	Low	Low	Low
Tsien et al., 2023 [13]	Low	Serious	Moderate	Low	Serious	Moderate	Moderate

**Table 3:** Adverse effects of BV alone.

Author	Diarrhea	Hypophosphatemia	Nausea and Vomiting	Headache	Fatigue	Constipation	Thrombocytopenia	Neutropenia	Lymphopenia	Anemia	High Blood Pressure
Field et al., 2015 [19]	24% (15/62)	Not reported	39% (24/62)	Not reported	84% (52/62)	29% (18/62)	23% (14/62)	6% (4/62)	Not reported	10% (6/62)	Not reported
Kelly et al., 2023 [20]	Not reported	Not reported	Not reported	Not reported	Not reported	Not reported	Not reported	Not reported	Not reported	Not reported	Not reported
Ellingson et al., 2018 [21]	BV alone was not used	BV alone was not used	BV alone was not used	BV alone was not used	BV alone was not used	BV alone was not used	BV alone was not used	BV alone was not used	BV alone was not used	BV alone was not used	BV alone was not used
Wirsching et al., 2021 [22]	BV alone was not used	BV alone was not used	BV alone was not used	BV alone was not used	BV alone was not used	BV alone was not used	BV alone was not used	BV alone was not used	BV alone was not used	BV alone was not used	BV alone was not used
Kickingereder et al., 2020 [23]	Not reported	Not reported	Not reported	Not reported	Not reported	Not reported	Not reported	Not reported	Not reported	Not reported	Not reported
Herrlinger et al., 2016 [24]	BV alone was not used	BV alone was not used	BV alone was not used	BV alone was not used	BV alone was not used	BV alone was not used	BV alone was not used	BV alone was not used	BV alone was not used	BV alone was not used	BV alone was not used
Gilbert et al., 2014 [25]	Not reported	Not reported	0.66% (2/303)	Not reported	2.3% (7/303)	Not reported	10.2% (31/303)	7.3% (22/303)	10.5% (32/303)	0.66% (2/303)	Not reported
Tsien et al., 2023 [13]	Not reported	Not reported	Not reported	Not reported	Not reported	Not reported	Not reported	Not reported	Not reported	Not reported	42.2% (24/57)
Friedman et al., 2009 [11]	1.2% (1/84)	Not reported	Not reported	36.9% (31/84)	45.2% (38/84)	Not reported	Not reported	1.2% (1/84)	2.4% (2/84)	Not reported	29.8% (30/84)
Nayak et al., 2021 [12]	BV alone was not used	BV alone was not used	BV alone was not used	BV alone was not used	BV alone was not used	BV alone was not used	BV alone was not used	BV alone was not used	BV alone was not used	BV alone was not used	BV alone was not used
Puduvalli et al., 2020 [15]	Not reported	Not reported	5.2% (2/38)	5.2% (2/38)	2.6% (1/38)	0% 0/38	Not reported	Not reported	2.6% (1/38)	Not reported	23.7 (9/38)
Weathers et al., 2016 [17]	Not reported	Not reported	Not reported	Not reported	11.1% (4/36)	Not reported	0% (0/36)	2.8% (1/36)	22.2% (8/36)	Not reported	5.6% (2/36)
Wirsching et al., 2018 [18]	BV alone was not used	BV alone was not used	BV alone was not used	BV alone was not used	BV alone was not used	BV alone was not used	BV alone was not used	BV alone was not used	BV alone was not used	BV alone was not used	BV alone was not used
Lee et al., 2020 [14]	Not reported	14% (8/57)	24.6% (14/57) *	57.9% (33/57)	66.7% (38/57)	Not reported	Not reported	Not reported	Not reported	Not reported	42.1% (24/57)
Reardon et al., 2020 [16]	1.2% (2/165)	not reported	5.5% (9/165) *	4.8% (8/165)	13.9% (23/165)	not reported	not reported	not reported	not reported	not reported	22.4% (37/165)
Total percentage in the articles that reported the effects	5.8%	14%	9.2%	36.87%	24.1%	29%	12.32%	5.77%	9.32%	2.19%	32.72%

* Just nausea.

**Table 4:** Adverse effects of BV + other modalities.

Author	BV + X	Diarrhea	Hypophosphatemia	Nausea and Vomiting	Headache	Fatigue	Constipation	Thrombocytopenia	Neutropenia	Lymphopenia	Anemia	High Blood Pressure
Field et al., 2015 [19]	BV + Carboplatin	26% (15/58)	Not reported	50% (29/58)	Not reported	86% (50/58)	45% (26/58)	55% (32/58)	24% (14/58)	Not reported	28% (16/58)	Not reported
Kelly et al., 2023 [20]	BV + Denifanstat	8% (2/25)	Not reported	8% (2/25) (only vomiting)	12% (3/25)	0.28% (7/25)	0.12% (3/25)	4% (1/25)	Not reported	Not reported	Not reported	52% (13/25)
Ellingson et al., 2018 [21]	BV + Radiotherapy + Temozolomide	Not reported	Not reported	Not reported	Not reported	Not reported	Not reported	Not reported	Not reported	Not reported	Not reported	Not reported
Wirsching et al., 2021 [22]	BV + Hypofractionated Radiotherapy	Not reported	Not reported	Not reported	Not reported	Not reported	Not reported	Not reported	Not reported	Not reported	Not reported	Not reported
Kickingereder et al., 2020 [23]	BV + Lomustina	Not reported	Not reported	Not reported	Not reported	Not reported	Not reported	Not reported	Not reported	Not reported	Not reported	Not reported
Herrlinger et al., 2016 [24]	BV + Radiotherapy + Irinotecan	Not reported	Not reported	4.2%	Not reported	Not reported	Not reported	Not reported	Not reported	Not reported	Not reported	Not reported
Gilbert et al., 2014 [25]	BV + Radiotherapy + Temozolomide	Not reported	Not reported	0.66% (2/303 during chemotherapy) 4% (11/260 during adjuvant treatment)	Not reported	2% (7/303 during chemotherapy) 13% (34/260 during adjuvant treatment)	Not reported	10% (31/303 during chemotherapy) 11% (29/260 during adjuvant treatment)	7% (22/303 during chemotherapy) 10% (26/250 during adjuvant treatment)	11% (32/303 during chemotherapy) 13% (34/260 during adjuvant treatment)	0.66% (2/303 during chemotherapy) 2% (6/260 during adjuvant treatment).	1.32% (4/303 during chemotherapy) 4% (11/260 during adjuvant treatment)
Tsien et al., 2023 [13]	BV + Radiotherapy	Not reported	Not reported	Not reported	Not reported	Not reported	Not reported	Not reported	Not reported	Not reported	Not reported	Not reported
Friedman et al., 2009 [11]	BV + Irinotecan	74.7% (59/79)	Not reported	67.1% * (53/79)	Not reported	75.9% (60/79)	Not reported	Not reported	8.9% (7/79)	7.6% (6/79)	Not reported	26.6% (21/79)
Nayak et al., 2021 [12]	BV + Pembrolizumab	6% (3/50)	Not reported	Not reported	16% (8/50)	18% (9/50)	Not reported	Not reported	Not reported	4% (2/50)	Not reported	50% (25/50)
Puduvalli et al., 2020 [15]	BV + Vorinostat (Suberoylanidized Acid)	Not reported	Not reported	0.0% (0/47) nausea and 0.0% (0/47) vomiting	2.1% (1/47)	4% (2/47)	2.1% (1/47)	Not reported	Not reported	4% (2/47)	Not reported	13% (6/47)
Weathers et al., 2016 [17]	BV + Lomustina	Not reported	Not reported	Not reported	Not reported	3% (1/35)	Not reported	23% (8/35)	14% (5/35)	46% (16/35)	Not reported	0.03% (1/35)
Wirsching et al., 2018 [18]	BV + Radiotherapy	Not reported	Not reported	Not reported	8% (4/50)	20% (10/50)	Not reported	Not reported	Not reported	Not reported	Not reported	26% (13/50)
Lee et al., 2020 [14]	BV + Trebananib	Not reported	8.6% (5/58)	13.8% (8/58)	34.5% (20/58)	63.8% (37/58)	Not reported	Not reported	Not reported	Not reported	Not reported	44.8% (26/58)
Reardon et al., 2020 [16]	-	-	-	-	-	-	-	-	-	-	-	-
Total percentage in the articles that reported the effects	-	15	8.6	13.37	15.65	22.48	23	14.83	10.2	11.88	3.86	13.2

* Just nausea.

**Table 5:** Adverse effects of BV.

Author	Diarrhea	Anorexia	Nausea and Vomiting	Headache	Fatigue	Thrombosis	Thrombocytopenia	Neutropenia	Lymphopenia	Leukopenia	Anemia	High Blood Pressure	Proteinuria	Constipation
Denda et al., 2016 [29]	12/48 25%	28/48 58%	24/48 50%, only nausea 10/48 21%, vomiting	**Not reported**	34/48 71%	2/48 4%	27/48 56%	32/48 67	**Not reported**	**Not reported**	44/48 92%	11/48 23%	4/48 8%	**Not reported**
Garcia et al., 2008 [30]	**Not reported**	**Not reported**	2/70 2.9%, only nausea 3/70 4.3%, vomiting	**Not reported**	6/70 8.6%	3/70 4.3%	**Not reported**	**Not reported**	14/70 20%	2/70 2.9%	**Not reported**	11/70 15.7%	3/70 4.3%	3/70 4.3%
Dellapasqua et al., 2008 [31]	16/46 34.8%	2/46 4.3%	24/46 52.2%, only nausea 8/46 17.4%, vomiting	**Not reported**	**Not reported**	**Not reported**	7/46 15.2%	15/46 32.6%	**Not reported**	27/46 58.7%	8/46 17.4%	22/46 47.8%	15/46 32.6%	17/46 37%
Kim et al., 2008 [32]	**Not reported**	**Not reported**	**Not reported**	**Not reported**	**Not reported**	**Not reported**	**Not reported**	**Not reported**	**Not reported**	**Not reported**	**Not reported**	**Not reported**	**Not reported**	**Not reported**
Dashti et al., 2022 [33]	**Not reported**	**Not reported**	2/10 20%	5/10 50%	**Not reported**	**Not reported**	**Not reported**	**Not reported**	**Not reported**	**Not reported**	**Not reported**	**Not reported**	**Not reported**	**Not reported**
Spitzer et al., 2008 [34]	**Not reported**	**Not reported**	**Not reported**	**Not reported**	**Not reported**	**Not reported**	**Not reported**	**Not reported**	**Not reported**	**Not reported**	**Not reported**	**Not reported**	**Not reported**	**Not reported**
Kudoh et al., 2011 [35]	4/30 13.3%	**Not reported**	12/30 40%, only nausea 5/30 16.7%, vomiting	**Not reported**	5/30 16.7%	**Not reported**	1/30 3.3%	7/30 23.3%	**Not reported**	13/30 43.3%	**Not reported**	7/30 23.3%	**Not reported**	10/30 33.3%
Tewari et al., 2023 [36]	**Not reported**	**Not reported**	**Not reported**	**Not reported**	**Not reported**	**Not reported**	**Not reported**	**Not reported**	**Not reported**	**Not reported**	**Not reported**	**Not reported**	**Not reported**	**Not reported**
Dupuis-Girod et al., 2014 [37]	**Not reported**	**Not reported**	**Not reported**	**Not reported**	**Not reported**	**Not reported**	**Not reported**	**Not reported**	**Not reported**	**Not reported**	**Not reported**	**Not reported**	**Not reported**	**Not reported**
Kennedy et al., 2018 [38]	**Not reported**	**Not reported**	**Not reported**	**Not reported**	**Not reported**	**Not reported**	**Not reported**	**Not reported**	**Not reported**	**Not reported**	**Not reported**	**Not reported**	**Not reported**	**Not reported**
Takemura et al., 2018 [39]	1/18 5.5%	**Not reported**	4/18 22.2%, only nausea	**Not reported**	**Not reported**	**Not reported**	1/18 5.5%	11/18 61.1%	**Not reported**	10/18 55.5%	**Not reported**	**Not reported**	**Not reported**	**Not reported**

## Supplementary Materials

The following supporting information can be downloaded at: https://www.mdpi.com/article/10.3390/ph18060795/s1. Table S1: Details of the search strategy. Table S2: Excluded studies and the reasons for their exclusion. References [54,55,56,57,58,59,60,61,62,63,64,65] are cited in the Supplementary Materials.
