# Supplementary material for: The Adverse Effects and Use of Bevacizumab in Patients with Glioblastoma: A Systematic Review and Meta-Analysis
Source: Pharmaceuticals (Basel). 2025 May 25;18(6):795. doi: 10.3390/ph18060795 (PMC12196191; doi:10.3390/ph18060795)
Supplement: Supplementary file 1 [file pharmaceuticals-18-00795-s001.zip › pharmaceuticals-3598751-supplementary.pdf]

Supplementary Table S1: Details of the search strategy

Database	Search strategy	Results	
		**20-10-24**	**20-11-2024**
Medline	("bevacizumab"[Title/Abstract] AND "glioblastoma multiform"[Title/Abstract]) OR "glioblastoma"[Title/Abstract]) AND (clinicaltrial[Filter] OR randomizedcontrolledtrial[Filter])	1718	1720
Wos	("bevacizumab"[Title/Abstract] AND "glioblastoma multiform"[Title/Abstract]) OR "glioblastoma"	110	110
CINAHL	("bevacizumab"[Title/Abstract] AND "glioblastoma multiform"[Title/Abstract]) OR "glioblastoma"	10	10
SCOPUS	("bevacizumab"[Title/Abstract] AND "glioblastoma multiform"[Title/Abstract]) OR "glioblastoma"	100	100
Google scholar	("bevacizumab"[Title/Abstract] AND "glioblastoma multiform"[Title/Abstract]) OR "glioblastoma"	35	35
	Total	1973	1975

* All searches were carried out on December 20, 2024.

Supplementary Table S2: Excluded studies and the reasons for their exclusion.

N°	Reference	Reason
1	Finn RS, Qin S, Ikeda M, Galle PR, Ducreux M, Kim TY, Kudo M, Breder V, Merle P, Kaseb AO, Li D, Verret W, Xu DZ, Hernandez S, Liu J, Huang C, Mulla S, Wang Y, Lim HY, Zhu AX, Cheng AL; IMbrave150 Investigators. Atezolizumab plus Bevacizumab in Unresectable Hepatocellular Carcinoma. N Engl J Med. 2020 May 14;382(20):1894-1905. doi: 10.1056/NEJMoa1915745. PMID: 32402160.	Another cancer region treated BV
2	Cheng AL, Qin S, Ikeda M, Galle PR, Ducreux M, Kim TY, Lim HY, Kudo M, Breder V, Merle P, Kaseb AO, Li D, Verret W, Ma N, Nicholas A, Wang Y, Li L, Zhu AX, Finn RS. Updated efficacy and safety data from IMbrave150: Atezolizumab plus bevacizumab vs. sorafenib for unresectable hepatocellular carcinoma. J Hepatol. 2022 Apr;76(4):862-873. doi: 10.1016/j.jhep.2021.11.030. Epub 2021 Dec 11. PMID: 34902530.	Another cancer region treated BV
3	Qin S, Chen M, Cheng AL, Kaseb AO, Kudo M, Lee HC, Yopp AC, Zhou J, Wang L, Wen X, Heo J, Tak WY, Nakamura S, Numata K, Uguen T, Hsiehchen D, Cha E, Hack SP, Lian Q, Ma N, Spahn JH, Wang Y, Wu C, Chow PKH; IMbrave050 investigators. Atezolizumab plus bevacizumab versus active surveillance in patients with resected or ablated high-risk hepatocellular carcinoma (IMbrave050): a randomised, open-label, multicentre, phase 3 trial. Lancet. 2023 Nov 18;402(10415):1835-1847. doi: 10.1016/S0140-6736(23)01796-8. Epub 2023 Oct 20. PMID: 37871608.	Another cancer region treated BV

4	Prager GW, Taieb J, Fakih M, Ciardiello F, Van Cutsem E, Elez E, Cruz FM, Wyrwicz L, Stroyakovskiy D, Pápai Z, Poureau PG, Liposits G, Cremolini C, Bondarenko I, Modest DP, Benhadji KA, Amellal N, Leger C, Vidot L, Tabernero J; SUNLIGHT Investigators. Trifluridine-Tipiracil and Bevacizumab in Refractory Metastatic Colorectal Cancer. N Engl J Med. 2023 May 4;388(18):1657-1667. doi: 10.1056/NEJMoa2214963. PMID: 37133585.	Another cancer region treated BV
5	Ray-Coquard, I., Pautier, P., Pignata, S., Pérol, D., González-Martín, A., Berger, R., Fujiwara, K., Vergote, I., Colombo, N., Mäenpää, J., Selle, F., Sehouli, J., Lorusso, D., Guerra Alía, E. M., Reinthaller, A., Nagao, S., Lefeuvre-Plesse, C., Canzler, U., Scambia, G., Lortholary, A., … PAOLA-1 Investigators (2019). Olaparib plus Bevacizumab as First-Line Maintenance in Ovarian Cancer. *The New England journal of medicine*, *381*(25), 2416–2428. https://doi.org/10.1056/NEJMoa1911361	Another cancer region treated BV
6	Tewari KS, Burger RA, Enserro D, Norquist BM, Swisher EM, Brady MF, Bookman MA, Fleming GF, Huang H, Homesley HD, Fowler JM, Greer BE, Boente M, Liang SX, Ye C, Bais C, Randall LM, Chan JK, Ferriss JS, Coleman RL, Aghajanian C, Herzog TJ, DiSaia PJ, Copeland LJ, Mannel RS, Birrer MJ, Monk BJ. Final Overall Survival of a Randomized Trial of Bevacizumab for Primary Treatment of Ovarian Cancer. J Clin Oncol. 2019 Sep 10;37(26):2317-2328. doi: 10.1200/JCO.19.01009. Epub 2019 Jun 19. PMID: 31216226; PMCID: PMC6879307.	Another cancer region treated BV
7	Oaknin A, Gladieff L, Martínez-García J, Villacampa G, Takekuma M, De Giorgi U, Lindemann K, Woelber L, Colombo N, Duska L, Leary A, Godoy-Ortiz A, Nishio S, Angelergues A, Rubio MJ, Fariñas-Madrid L, Yamaguchi S, Lorusso D, Ray-Coquard I, Manso L, Joly F, Alarcón J, Follana P, Romero I, Lebreton C, Pérez-Fidalgo JA, Yunokawa M, Dahlstrand H, D'Hondt V, Randall LM; ENGOT-Cx10–GEICO 68-C–JGOG1084–GOG-3030 Investigators. Atezolizumab plus bevacizumab and chemotherapy for metastatic, persistent, or recurrent cervical cancer (BEATcc): a randomised, open-label, phase 3 trial. Lancet. 2024 Jan 6;403(10421):31-43. doi: 10.1016/S0140-6736(23)02405-4. Epub 2023 Dec 1. PMID: 38048793.	Another cancer region treated BV
8	Casadei-Gardini, A., Rimini, M., Tada, T., Suda, G., Shimose, S., Kudo, M., Cheon, J., Finkelmeier, F., Lim, H. Y., Rimassa, L., Presa, J., Masi, G., Yoo, C., Lonardi, S., Tovoli, F., Kumada, T., Sakamoto, N., Iwamoto, H., Aoki, T., Chon, H. J., … Cucchetti, A. (2023). Atezolizumab plus bevacizumab versus lenvatinib for unresectable hepatocellular carcinoma: a large real-life worldwide population. *European journal of cancer (Oxford, England : 1990)*, *180*, 9–20. https://doi.org/10.1016/j.ejca.2022.11.017	Another cancer region treated BV
9	Tewari, K. S., Sill, M. W., Penson, R. T., Huang, H., Ramondetta, L. M., Landrum, L. M., Oaknin, A., Reid, T. J., Leitao, M. M., Michael, H. E., DiSaia, P. J., Copeland, L. J., Creasman, W. T., Stehman, F. B., Brady, M. F., Burger, R. A., Thigpen, J. T., Birrer, M. J., Waggoner, S. E., Moore, D. H., … Monk, B. J. (2017). Bevacizumab for advanced cervical cancer: final overall survival and adverse event analysis of a randomised, controlled, open-label, phase 3 trial (Gynecologic Oncology Group 240). *Lancet (London, England)*, *390*(10103), 1654–1663. https://doi.org/10.1016/S0140-	Another cancer region treated BV

	6736(17)31607-0	
10	McDermott, D. F., Huseni, M. A., Atkins, M. B., Motzer, R. J., Rini, B. I., Escudier, B., Fong, L., Joseph, R. W., Pal, S. K., Reeves, J. A., Sznol, M., Hainsworth, J., Rathmell, W. K., Stadler, W. M., Hutson, T., Gore, M. E., Ravaud, A., Bracarda, S., Suárez, C., Danielli, R., … Powles, T. (2018). Clinical activity and molecular correlates of response to atezolizumab alone or in combination with bevacizumab versus sunitinib in renal cell carcinoma. *Nature medicine*, *24*(6), 749–757. https://doi.org/10.1038/s41591-018-0053-3	Another cancer region treated BV
11	Eroğlu, Y., Baykara, M., Perçinel Yazici, İ., Utku Yazici, K., & Kürşad Poyraz, A. (2022). Evaluation of the corpus callosum using magnetic resonance imaging histogram analysis in autism spectrum disorder. *The neuroradiology journal*, *35*(6), 751–757. https://doi.org/10.1177/19714009221097507	Another cancer region treated BV
12	Vergote I, Van Nieuwenhuysen E, O'Cearbhaill RE, Westermann A, Lorusso D, Ghamande S, Collins DC, Banerjee S, Mathews CA, Gennigens C, Cibula D, Tewari KS, Madsen K, Köse F, Jackson AL, Boere IA, Scambia G, Randall LM, Sadozye A, Baurain JF, Gort E, Zikán M, Denys HG, Ottevanger N, Forget F, Mondrup Andreassen C, Eaton L, Chisamore MJ, Viana Nicacio L, Soumaoro I, Monk BJ. Tisotumab Vedotin in Combination With Carboplatin, Pembrolizumab, or Bevacizumab in Recurrent or Metastatic Cervical Cancer: Results From the innovaTV 205/GOG-3024/ENGOT-cx8 Study. J Clin Oncol. 2023 Dec 20;41(36):5536-5549. doi: 10.1200/JCO.23.00720. Epub 2023 Aug 31. PMID: 37651655; PMCID: PMC10730069.	Another cancer region treated BV
